# An analysis of dialogue repair in virtual assistants

**DOI:** 10.3389/frobt.2024.1356847

**Published:** 2024-11-11

**Authors:** Matthew Galbraith

**Affiliations:** Department of Translation and Language Sciences, Universitat Pompeu Fabra, Barcelona, Spain

**Keywords:** conversational user interface, interactional language, conversational repair, conversation analysis, dialogue repair, virtual assistants, human-computer interaction, human-robot interaction

## Abstract

Conversational user interfaces have transformed human-computer interaction by providing nearly real-time responses to queries. However, misunderstandings between the user and system persist. This study explores the significance of interactional language in dialogue repair between virtual assistants and users by analyzing interactions with Google Assistant and Siri in both English and Spanish, focusing on the assistants’ utilization and response to the colloquial other-initiated repair strategy “*huh?*”, which is prevalent as a human-human dialogue repair strategy. Findings revealed ten distinct assistant-generated repair strategies, but an inability to replicate human-like strategies such as “*huh?*”. Despite slight variations in user acceptability judgments among the two surveyed languages, results indicated an overall hierarchy of preference towards specific dialogue repair strategies, with a notable disparity between the most preferred strategies and those frequently used by the assistants. These findings highlight discrepancies in how interactional language is utilized in human-computer interaction, underscoring the need for further research on the impact of interactional elements among different languages to advance the development of conversational user interfaces across domains, including within human-robot interaction.

## 1 Introduction

A hallmark of human-human interaction (HHI) is the use of interactional language which encompasses the complex dynamics of linguistic and paralinguistic features employed by humans in dialogues including turn-taking, politeness, dialogue repair mechanisms, and prosodic cues (Ginzburg, 2012; Levinson, 2019; Wiltschko, 2021). These meta-features define the conversations that humans hold every day and, increasingly, when speaking with voice assistants and robotic companions (Chaves and Gerosa, 2021). Examining the use of interactional language in human-computer interaction (HCI) and human-robot interaction (HRI) offers a unique view into the functionality of communication strategies with non-human interlocutors.

Within interactional language, dialogue repair refers to collaborative strategies used to address and resolve misunderstandings during conversations. Dialogue repair manifests in various forms, such as requests for clarification, repetition of misunderstood information, or paraphrasing to confirm mutual comprehension (Ashktorab et al., 2019; Cho and Larke, 2010; Kendrick, 2015). These actions serve to restore the progression of the dialogue and ensure that the intended message is accurately conveyed and understood. Repair as a part of interaction has been the subject of extensive study. Schegloff et al. (1977) findings were a turning point in repair systematization, highlighting key indicators that were present in repair sequences: the *repairable*, the *repair initiator* and the *repair outcome*. These repairs occur in either self-repair or other-initiated repair (OIR) forms. Self-repair involves a speaker acknowledging an error in their speech and taking action to correct it, whereas OIR involves the listener identifying the error and instigating the correction process. Schegloff et al. (1977) identify five categories of repair.

1. Unspecified Repair–Includes a repair initiator such as “*huh?*” or “*what?*”; this type of strategy does not specify the trouble source, and these repair initiations usually yield a repetition of the trouble source turn.
2. Interrogative Repair–Includes a single question word such as “*who?*”, “*where?*”, or “*when?*” as a repair initiator; this type of strategy specifies a trouble source of the prior turn.
3. Partial Repeat Plus a Question Word–Includes a question word with a partial repeat of the trouble source turn.
4. Partial Repeat Repair–The trouble source turn is partially repeated and used for repair initiation.
5. Understanding Check Repair–The initiator is “*you mean…?*” plus a possible understanding of prior turn; here, the listener initiates the repair at the trouble source by giving an alternate understanding of the trouble source, then the speaker completes the repair in the subsequent turn.

Clark (1996) offers a foundational perspective on the coordination of actions, responses, and interpretations that are essential for achieving mutual understanding in dialogue. The risk of misunderstanding emerges from this complex coordination, requiring mechanisms for repair. Clark (1996) concepts of *joint construals* and *joint closure* in effective communication detail a scenario in which both parties work toward a mutual belief that their intended messages have been successfully communicated. These processes are prone to errors due to varying interpretations shaped by individual perspectives and contexts, underscoring the need for repair to maintain the flow of conversation. Purver (2004) expands upon this by documenting how misunderstandings manifest and are addressed, emphasizing that clarification requests serve as a critical tool for managing communication breakdowns due to their utterance-anaphoric nature, which refers back to and queries specific aspects of the problematic utterance. Purver (2004) builds on the work of Ginzburg and Cooper (2004) by describing how clarification requests arise in dialogue, highlighting the layered nature of dialogue repair, wherein conventional clarification requests signal varying degrees of comprehension breakdown.

Expanding on the significance of dialogue repair, many studies have emphasized the significance of OIR, noting its occurrence at an average of 1.4 times per minute across various languages (Dingemanse et al., 2015; Enfield et al., 2012; Levinson, 2019). This high frequency demonstrates the pervasiveness of miscommunications in language, underscoring the importance of having strategies that can repair them. The use of “*huh?*” has garnered considerable attention, particularly with Dingemanse et al. (2013) investigation (building upon work by Enfield et al. (2012)), which explores the cross-linguistic similarities of “*huh?*” as an OIR initiator. Dingemanse et al. (2015) continuation of this study examines this phenomenon using data collected from multiple languages originating from diverse linguistic families. The findings reveal that “*huh?*” has a functional role in initiating repairs in the languages studied, highlighting its pragmatic value and differentiating it from being a backchannel which merely serves to “indicate [that] the addressee is following [the flow of the dialogue] and acknowledging the content of the preceding utterance without claiming a turn” (Wiltschko, 2021). These instances of “*huh?*” also tend to share a similar rising intonational contour, suggesting a universal prosodic pattern that coincides with Schegloff et al. (1977) observations of rising intonation being a feature of OIR initiators. The universality of “*huh?*”, bolstered by its pragmatic role in managing communication breakdowns, make it a prime candidate for further inquiry in interactional language studies in HCI/HRI and serve as the motivation for its use in this investigation.

Previous research has primarily focused on interactional language as it pertains to HHI; its application to HCI and HRI is relatively new and presents a unique set of challenges and opportunities. While conventional knowledge describes conversational user interfaces (CUIs) as striving to emulate human-like interactions, they often struggle to capture the subtleties of human expression, resulting in a limited and less satisfactory output (Chaves and Gerosa, 2021; Motta and Quaresma, 2022). CUIs, ranging from virtual assistants like Apple’s Siri and Google’s Google Assistant (two of the most popular as noted by Hoy, 2018) to robotic personal companions, have become an integral part of daily life, revolutionizing HCI and HRI. The increasing ubiquity of voice-based assistants has sparked a growing interest in understanding how to improve these systems, prompting the need for more expansive research. Despite this expansion, however, research involving CUIs functioning in languages other than English has received less attention. Spanish, one of the two languages tested in the present study, boasts a substantial number of speakers worldwide but can lack the necessary attention and resources to build robust linguistic data for complex systems (Bender, 2019; Gutiérrez-Fandiño et al., 2022; Nicholas and Bhatia, 2023). Allwood (2000) extends this criticism of a lack of cultural sensitivity to conversational analysis itself (which the present study draws upon in its analysis), specifically that it fails to consider variations in turn-taking and other conversational features across different cultures. This critique likewise motivates the current research to address this limitation by comparing conversational analysis-based assessments of OIR in English and Spanish. Indeed, a monolingual focus on English when developing CUIs becomes a potential failure point that can have widespread consequences throughout nearly every part of their language processing pipelines, from speech recognition to semantic decoding, natural language generation, and more (Bender, 2009; Wiggers, 2019).

Like in HHI, interactions between users and CUIs are susceptible to recurring errors—the mechanical interlocutor may misconstrue queries, misinterpret input due to speaker accent, or become confused by references to previous turns (Ashktorab et al., 2019). Ashktorab et al. (2019) explain that resolving communication breakdowns in HCI is necessary to avoid a negative user response as these miscommunications, even when they are repaired by recovery strategies, decrease the user’s satisfaction, trust, and willingness to continue using the system. Motta and Quaresma (2022) conducted research on this phenomenon in virtual assistants, categorizing errors that users encountered while interacting with the assistants (though these errors were incidental and not purposefully induced by users):

• Different Task—The assistant performs an activity or task other than the requested task.
• Wrong Information—The assistant ascribes incorrect details to the requested task.
• Input Failure—The assistant does not capture any part of the user’s command.
• Interruption—The assistant stops capturing input halfway through the user’s commands.
• Misrecognition—The assistant misrecognizes one or more words in the input.
• Request for Manual Interaction—The assistant asks the user to interact manually to add information, save, or cancel events. This was considered an error type as users consider requests for manual interaction as a nuisance.
• Error Messages—The assistant explicitly tells the user that an error has occurred (e.g., “Sorry, I didn’t understand”) or that it cannot perform the task.
• Instructions—The assistant explicitly tells the user how to proceed in case of error or offer directions to move the interaction forwards (e.g., “Say ‘yes’ to save”). Questions (e.g., “What is the event’s title?”) and confirmations (e.g., “I created the event”) were considered indirect instructions as they provide cues to advance interactions.

While the present investigation seeks to determine the significance of interactional language in dialogues with virtual assistant platforms, they are not the only CUIs to suffer from breakdowns in communication—these challenges are analogous to need for repair faced by robots operating in dynamic, real-world environments where seamless interaction with humans is paramount (Stommel et al., 2022; Trott and Rossano, 2017). The study of dialogue repair mechanisms in virtual assistants intersects with the field of robotics, especially in the progress of HRI systems which have been described as “rigid” and “arguably asocial” at times (Stommel et al., 2022). By leveraging insights from this research on dialogue repair in virtual assistants, researchers can develop adaptive strategies for robotic systems to recover from communication failures, enhance user experience, and ultimately enable more effective and natural human-robot collaboration. Indeed, bridging virtual assistants and robotics provides an opportunity to improve not only the robustness of HRI systems, but to also contribute to the broader goal of creating intelligent, socially-aware machines capable of fluent and contextually-appropriate interactions.

The following details the main research questions driving this investigation, along with corresponding hypotheses posited as possible outcomes.

• RQ1: How do Google Assistant and Siri handle dialogue repair when the user is initiating OIR by using “*huh?*” ?

Due to the overall absence of integration of interactional language in CUIs, it is likely that these systems will encounter challenges in accurately parsing and handling “*huh?*” from the user when used as a request for OIR.

• RQ2: Is it possible to elicit the OIR strategy “*huh?*” when in a dialogue with Google Assistant and Siri? If not, which repair strategies are used in its stead?

It is unlikely that assistants will employ this repair strategy due to the lack of development that the systems have regarding interactional language specific to dialogue repair. Instead, it is assumed that the assistants will rely on more precise and formal language to fulfill the user’s request while sacrificing the humanistic quality of interactional language.

• RQ3: Do dialogue repair strategies used by Google Assistant and Siri vary between English and Spanish? If so, how?

Given the comparatively limited developmental focus on non-English languages in virtual assistants, it is expected that a discrepancy will arise in the frequency and quality of the strategies employed in English and Spanish across both assistants.

• RQ4: How acceptable do users find the dialogue repair strategies produced by Google Assistant and Siri?

Taking into account the task-based nature of the requests given to the assistants, users will likely strongly favor strategies that prioritize trying to fulfill requests at any cost, rather than strategies that attempt to sidestep or outright ignore the misunderstanding.

It is important to note that any hypotheses regarding the use of interactional language in CUIs are inherently tentative, as the exploration of this phenomenon in machines is still in its early stages. However, given the research questions presented above and taking into account previous investigations regarding CUIs (Ashktorab et al., 2019; Galbraith and Martínez, 2021; Motta and Quaresma, 2022), the corresponding hypotheses were generated for this pilot study.

## 2 Materials and methods

### 2.1 Overview

Two tests, Task A and Task B, were performed to assess the proficiency of Google Assistant and Siri in generating an OIR-like response (Task A) and in reacting to the OIR initiator “*huh?*” (Task B). Both tasks required speech-based interaction between the researcher and the virtual assistants, followed by transcription of the dialogues for analysis. The tasks were conducted in both American English and Castilian Spanish by the researcher, an individual fluent in both languages and dialects. Google Assistant tasks were performed utilizing a Samsung Galaxy S21 running Android 14 with the integrated Google application (version 14.20.18) which includes Assistant, while Siri interactions were accomplished with an iPhone SE running iOS 16.3.1^1^. The assistants in both Task A and Task B were configured to communicate in the specific target language under examination.

In Task A and Task B the researcher introduced queries to the assistants that were purposefully constructed to cover tasks (which are parsed by the assistants to identify the underlying purpose of the request, known as “intents”) within thematic domains that are common among these assistants: setting a reminder, getting directions, playing a song, retrieving information about a topic, sending messages, starting a phone call, and getting the weather forecast. An exception to this was in Task A which introduced a non-lexical (henceforth: *unintelligible*) phrase in isolation to the assistant, e.g., “*Ok Google, {unintelligible phrase}*”. This query was presented to the assistant to explore the repair strategy that would be returned in the absence of any additional information confirming the specific intent or purpose behind the request. All queries used in these tasks have been provided in the Supplementary Materials.

A third task, Task C, was administered in the form of a survey to English (*n* = 50) and Spanish (*n* = 50) participants (for a total of 100 participants) using the online research platform Prolific. These surveys were distributed to participants who provided acceptability judgements for the dialogues created in Task A and Task B, with the goal of evaluating their preferences towards the repair strategies that the assistants utilized in those tasks.

### 2.2 Methodological considerations

#### 2.2.1 Acceptability judgements

Acceptability judgments have been used extensively in linguistic research to provide insights into a speaker’s perception of language beyond grammaticality (Bauer, 2014). The distinction between grammaticality and acceptability lies in the difference between what a grammar can produce (grammaticality) versus the practical usage that speakers find appropriate (acceptability) (Bauer, 2014; Chomsky, 1965). As such, acceptability is directly related to a speaker’s performance and reflects the naturalness and contextual appropriateness of a given linguistic construction (Chapman and Routledge, 2009). The use of these judgments as a valid metric for analysis is a widespread and well-accepted practice within linguistics—Sprouse et al. (2013) observe that, of the US-English articles published in *Linguistic Inquiry* between 2001 and 2010, 77% were based on some type of acceptability judgment.

In the present study, Likert scores were used as the primary metric to test acceptability. These scores have proven effective in quantifying acceptability and allow for the comparison of various conditions and effect sizes (Langsford et al., 2018). The emergence of these scores as a technique for evaluating acceptability judgments can be attributed to their high reliability, alignment with testing judgments, and their intuitive appeal to participants (Schütze and Sprouse, 2014; Sprouse et al., 2013; Langsford et al., 2018).

#### 2.2.2 Repair classification

This study assesses its findings using a framework of 10 dialogue repair strategies, as outlined in Table 1. Each category represents strategies observed when virtual assistants attempted to resolve miscommunications during dialogues with the researcher. This schema builds on Galbraith and Martínez (2021), which categorized strategies based on voice assistant outputs and repair strategies in HCI and HHI (Cho and Larke, 2010; Egbert, 1997; Liebscher and Dailey-O’Cain, 2003; Motta and Quaresma, 2022; Schegloff et al., 1977). The present work refines that framework, rephrasing some strategies for clarity and incorporating previously unaccounted behaviors. Galbraith and Martínez (2021) also introduces the concept of using interactional language in dialogue repair, which is fundamental to this study. Additionally, the present work expands on that prior study’s methodology by extending testing to multiple dialogue turns in Task B and gathering real user perspectives through surveys in Task C.

**TABLE 1:** Assistant dialogue repair strategies.

Shorthand	Name	Strategy Description and Example
Strategy 1	Partial Fulfillment	Attempts to fulfill requests given the information, excluding the unintelligible part. **Example:** User: Hey Assistant, I have an appointment at the dentist for *{unintelligible phrase}.* Assistant: I did not find any appointment at “dentist” in the next 3 months.
Strategy 2	Literal Interpretation	Takes a literal interpretation of the unintelligible part of the utterance and attempts to use the appropriate action to fulfill the request. **Example:** User: Hey Assistant, how can I get to the street *{unintelligible phrase}*? Assistant: I could not find any places matching “Street *{unintelligible phrase}*.”
Strategy 3	Misunderstanding	Fails to understand or mishears - sometimes proposes an error, may be accompanied by the system asking for the user to ask again. **Example:** User: Hey Assistant, play the song {*unintelligible phrase*}. Assistant: Uh oh, there’s a problem. Please, try again.
Strategy 4	Request Cancellation	Not capable of fulfilling requests (sometimes cancels the request due to this inability, or remains completely silent). **Example:** User: Hey Assistant, how long does it take from Balmes street to *{unintelligible phrase}*? Assistant: Sorry, I cannot give you traffic to that location.
Strategy 5	Clarification Request	Asks for the appropriate information that may include or may not include the unintelligible speech. **Example:** User: Hey Assistant, call *{unintelligible phrase}*. Assistant: Who would you like to call?
Strategy 6	Internet Search	Searches the internet for information that it attempts to parse from the unintelligible part of the phrase. **Example:** User: Hey Assistant, what is the capital of *{unintelligible phrase}*? Assistant: I found this on the web.
Strategy 7	User Data Utilization	Uses information stored about the user to fulfill requests. **Example:** User: Hey Assistant, how can I get to the street *{unintelligible phrase}*? Assistant: The best way to get to [user’s home address] is…
Strategy 8	App Suggestion	Opens (or proposes) an application (that may or may not be available on the user’s device) that the user can further use to ask the request again to better fulfill it. **Example:** User: Hey Assistant, play the new album of *{unintelligible phrase}*. Assistant: [Opens YouTube app].
Strategy 9	Instructions Provided	Gives the user a list of instructions (or the website to find the information) for the operation of the assistant. **Example:** User: Hey Assistant, *{unintelligible phrase}*. Assistant: [Gives instructions about possible actions with the assistant].
Strategy 10	Phonetic Transcription	Attempts to phonetically transcribe the unintelligible part of the utterance, but ultimately fails to provide any further action. **Example:** User: Hey Assistant, *{unintelligible phrase}*. Assistant: [No response, unintelligible phrase partially parsed].

The dialogue repair classification developed in Galbraith and Martínez (2021) and extended in this work was created with the intention of aligning with established theories of communication and repair, particularly those concerning the management of misunderstandings in dialogue. Building on Clark (1996) concepts of *joint construals* and *joint closure*, this classification reflects the essential coordination between dialogue participants aimed at achieving mutual understanding and closure in a dialogue. Specifically, the framework incorporates the use of clarification requests—key tools in resolving communication breakdowns that are highlighted by Purver (2004) work. The classification is also fundamentally based on the principles of Conversation Analysis, as established by Sacks et al. (1974), and it also incorporates the concept of progressivity, which emphasizes sustaining mutual understanding through turn-taking and maintaining the flow of conversation (Albert and de Ruiter, 2018). While virtual assistants’ strategies may differ from human methods, their categorization as repair strategies is justified by their focus on resolving communication breakdowns and maintaining dialogue continuity.

The classification framework used in this study was designed to categorize repair strategies based on the minimal actions an assistant could take to address a dialogue breakdown, with a consideration for whether these actions furthered the conversation or caused significant interruptions. The differentiation of strategies was grounded in the observed responses of the assistant when a repair was needed. Each strategy was then evaluated to determine whether the assistant’s action maintained the flow of dialogue, in line with the principles of progressivity and joint closure, or caused a disruption that stalled the conversation. This approach helped identify and categorize the strategies based on the assistants’ immediate and minimal reactions, providing a clear distinction between these actions and a glimpse into those that contributed to maintaining dialogue momentum or leading to its interruption. For example, most strategies were associated with distinct actions by the assistant that helped advance the conversation, aligning with the principle of progressivity. However, strategies like Strategy 4 [Request Cancellation] and Strategy 10 [Phonetic Transcription] either ended the dialogue flow or failed to contribute meaningfully to it, thereby interrupting the progression of the dialogue. By focusing on the assistant’s minimal actions and their impact on the dialogue, the framework effectively differentiates repair strategies based on assistants’ actions and their ability to either sustain or disrupt communication, reflecting the real-time decisions made by the assistant in response to dialogue repair needs.

#### 2.2.3 Assessing repair origin

**FIGURE 1:**
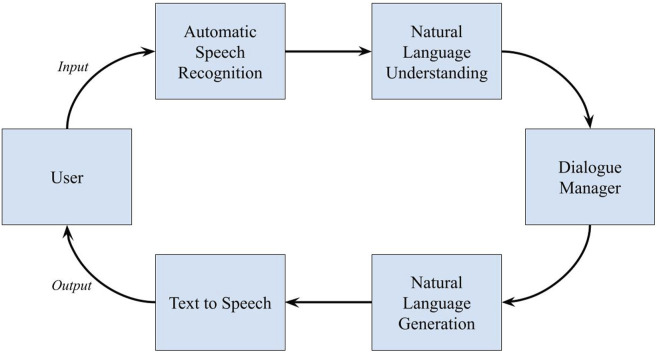
CUI dialogue processing pipeline.

The current investigation focuses on evaluating miscommunications as conveyed by the output of the dialogue manager, rather than emphasizing the origin of the failure from any specific stage in the processing pipeline. This pipeline includes several stages (depicted in Figure 1): user input in the form of audio, automatic speech recognition (ASR), semantic and syntactic analysis by the natural language understanding (NLU) module, the dialogue manager’s decision-making process, natural language generation (NLG), and finally text to speech audio synthesis. The dialogue manager undertakes crucial back-end processing including tracking dialogue context and selecting appropriate system actions, the result of which is transformed into a response to the user (Young, 2010). While failures of distinct types—either mishearing (ASR-based errors) or misunderstanding (NLU-based errors)—can occur in stages leading up to the information reaching the dialogue manager, it is the surface realization of the manager’s final output that this experiment sought to classify. This focus on the final output is due to the proprietary nature of the CUIs tested, which do not permit detailed examination of the dialogue management processes beyond the limited access provided by the developers, restricting the ability to identify the specific stage in the dialogue processing pipeline where a failure occurred. Principally, then, the output produced by the dialogue manager is the sole indication of the system’s final decision to the interactant; the user only has the information provided by the output to understand what the system has done, and consequently how they should respond. This can manifest in the user seeking a repair request if the result is infelicitous, or stopping the interaction if satisfied by the outcome. As such, these conditions force a pointed evaluation of the dialogue manager’s output rather than focusing solely on any particular failure point within the processing pipeline. Of note, this does not rule out the possibility of the dialogue manager expressly stating the source of the failure in its output—i.e., “*I did not hear you*.” However, while this phase may superficially seem to be directly indicative of a failure in the ASR portion of the pipeline, there is no explicit way of confirming this to be the case due to the obfuscated nature of the assistants tested. This output could be a response that either directly implicates an ASR failure or, more broadly, indicates a general misunderstanding. This reasoning serves as the impetus for placing, for example, repair strategies of “mishearing” into the same category of general misunderstanding within this analysis (Strategy 3 [Misunderstanding] in Table 1). Given this reasoning and the conversation analysis-based approach taken in the analysis of the observed dialogue repair strategies, the present research does not attempt to differentiate between ASR and NLU errors.

### 2.3 Task methodology

#### 2.3.1 Task A- assistant repair production

The initial test, Task A, aimed to answer RQ2/RQ3 by testing the assistants’ ability to produce repair initiators. This entailed the researcher purposefully introducing an unintelligible phrase embedded within a voice-based request to create a source for misinterpretation in the dialogue. The unintelligible phrase [t̪a.t̪a.ˈt̪a] was employed in Spanish, and its phonetic equivalent [tɑ. tɑ.ˈtɑ] in English. The selection of these phrases was intended to maintain a realistic phonetic realization within the phonemic inventories of the respective languages while avoiding any recognized lexical words.

This procedure is summarized below:

1. Initiate a speech dialogue with the assistant (Google Assistant/Siri) in the target language being tested (American English/Castilian Spanish) using the assistant’s wake word as a voice command (Siri - English “Hey Siri”/Spanish “Oye Siri”, Google Assistant - English “Ok Google”/Spanish “Ok Google^2^”).
2. When prompted, begin a query^3^ and include an intentionally unintelligible phrase ([t̪a.t̪a.ˈt̪a] in Spanish, [tɑ.tɑ.ˈtɑ] in English) instead of the core information needed by the assistant to fulfill the request.
3. Allow the assistant to audibly respond and record its output as text, classifying the repair strategies used according to type (utilizing the schema in Table 1).
4. Repeat steps 1-4, for 30 total queries (15 in English and 15 in Spanish) using the same queries made in English but adapted to Spanish.

Task A Example:

$\underset{“Play\;the\;song\;{\left\{unintelligible\;phrase\right\}}^{”}}{Researcher}\to$ $\underset{\left[Opens\;music\;app\right]}{Assistant}$

#### 2.3.2 Task B- assistant repair comprehension

The subsequent experiment, Task B, aimed to answer RQ1/RQ3 by testing the assistants’ ability to comprehend and manage the repair initiator “*huh?*” introduced by the researcher throughout the course of a voice-based dialogue. The experimental procedure entailed the researcher initiating a speech query designed so that the assistant would require further information to fulfill the request, prompting the assistant to respond by soliciting more details, i.e., *Researcher: “I need to set an alarm”* → *Assistant:* “*What time is the alarm for?*”. In the following dialogue turn, the researcher introduced the repair initiator “*huh?*” to which the assistant was prompted to respond. Upon receiving the assistant’s response to the repair, the researcher once again introduced the repair initiator “*huh?*”, eliciting a final response from the assistant.

This procedure is summarized below:

1. Initiate a speech dialogue with the assistant (Google Assistant/Siri) in the target language being tested (American English/Castilian Spanish) using the assistant’s wake word as a voice command (Siri - English “Hey Siri”/Spanish “Oye Siri”, Google Assistant - English “Ok Google”/Spanish “Ok Google”).
2. When prompted, begin a query^4^ that requires additional information to be fulfilled by the assistant.
3. Allow the assistant to solicit the additional information.
4. In response to this solicitation, have the researcher introduce the OIR initiator “*huh?*”.
5. Allow the assistant to respond and record its output, classifying the repair strategies used according to type (utilizing the schema in Table 1).
6. In response to the output by the assistant in step 5, have the researcher again introduce the OIR initiator “*huh?*”.
7. Allow the assistant to respond and record this final output as text, classifying the repair strategies used according to type (utilizing the schema in Table 1).
8. Repeat steps 1-7 for 20 total queries^5^ (10 in English and 10 in Spanish), using the same queries made in English but adapted to Spanish, replacing the English OIR initiator “*huh?*” with the Spanish equivalent, “*¿eh?*”.

Task B Example:

$\underset{“Set\;an\;alarm^{”}}{Researcher}$ → $\underset{“For\;what\;time{?}^{”}}{Assistant}$ → $\underset{“Huh{?}^{”}}{Researcher}$ → $\underset{“For\;what\;time{?}^{”}}{Assistant}$ → $\underset{“Huh{?}^{”}}{Researcher}$ → $\underset{“For\;what\;time{?}^{”}}{Assistant}$

#### 2.3.3 Task C - repair user evaluation

The final task, Task C, consisted of creating two surveys using the Qualtrics platform—one in American English and one in Castilian Spanish—that were distributed using the crowd-sourcing platform Prolific to 50 participants (*n* = 50) per language, for a total of 100 participants. The Prolific platform allowed for screening of candidates based on their linguistic background, which ensured that each test was taken by native English or Spanish speakers, respectively. Participants were also screened for age to conform to ethical guidelines, with all participants being between 18–65 years of age. These surveys were written to elicit acceptability judgements from participants as they viewed samples of dialogues taken from both Task A and Task B. The judgements provided were recorded using a five-point Likert scale with a range of: “completely unacceptable”, “somewhat unacceptable”, “neither acceptable nor unacceptable”, “somewhat acceptable”, and “completely acceptable” (and the equivalent in the Spanish version of the survey). For display purposes in the figures included in this paper, the five-point scale was collapsed into “unacceptable”, “neutral”, and “acceptable” categories, but the underlying analysis retained the full breadth of the original scale, with all five categories kept separate to ensure detailed examination of participant responses.

The dialogues presented to participants in these surveys (seen in Supplementary Table 3 of the attachment) were sourced from assistant responses from Task A and Task B. For each dialogue repair strategy, two dialogues were presented for a total of 40 individual dialogues—20 coming from Task A and 20 from Task B. For each of these two dialogues per repair strategy, one dialogue was sourced from each assistant when possible^6^. The dialogues were presented to users in two parts within the survey—one part consisting of 20 dialogues in randomized order derived from Task A, and another part consisting of 20 dialogues in randomized order derived from Task B.

## 3 Results

The findings from Task A, Task B, and the combined data are presented in the following sections. To explore the relationship between task, language, or assistant and the repair strategies generated, a Bayesian test of association was conducted using the *BayesFactor* package in R, applying a joint multinomial sampling approach (Morey et al., 2024). This Bayesian method was selected due to the categorical nature of the data and the challenges posed by limited sample sizes and uneven distributions. The flexibility of Bayesian tests in modeling relationships without requiring normality or linearity assumptions makes them suitable for this study (Miočević et al., 2020). The Bayesian test of association was chosen over the correlated t-test for its ability to evaluate relationships between categorical variables without relying on distributional assumptions. This choice aligns better with the categorical nature of the repair strategies and their frequencies observed in the data (Miočević et al., 2020). In contrast, the Bayesian correlated t-test, typically applied to continuous data, would not adequately address the categorical relationships in this study (Webb, 2021). All code used in this analysis is provided in Galbraith (2024).

All dialogue repair strategies in the following results are referenced as ‘Strategy # [Descriptive Name].’ The numerical identifier (Strategy #) aids in analysis and clarity within the figures, while the descriptive name improves readability and minimizes the need to cross-reference the full descriptions in Table 1.

### 3.1 Task A- results

**FIGURE 2:**
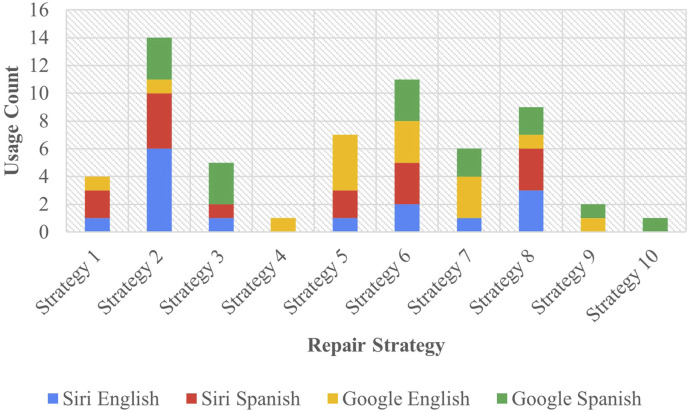
Task A results - assistant repair strategies.

In Task A, deliberately unintelligible input was introduced to the assistant, aiming to elicit a repair response similar to the interactional repair initiator “*huh?*”. The distinct assistant responses observed in this task are illustrated in Figure 2.

The most commonly observed repair approach was Strategy 2 [Literal Interpretation], implemented 14 times, making up 23.33% of all strategies employed in the task. Among these instances, Siri utilized this strategy 10 times, comprising 33% of the assistant’s total output, while Google employed it 4 times, constituting 13.33% of its total output. Strategy 2 [Literal Interpretation] accounted for 23.33% of all strategies used across both English and Spanish, with each language utilizing it 7 times equally. Following closely was Strategy 6 [Internet Search], utilized 11 times, and representing 18.33% of the total strategies used in the task. Specifically, Siri employed Strategy 6 [Internet Search] in five instances, accounting for 16.67% of its total output, while Google Assistant utilized it 6 times, representing 20% of its total output. This constituted 20% of overall Spanish usage and 16.67% of total English usage.

Strategy 4 [Request Cancellation] was the among the least utilized, appearing only once and accounting for 1.67% of the total strategy use. This strategy was exclusively used by Google in English, constituting 3.33% of all English strategies and 3.33% of all Google strategies. Similarly, Strategy 10 [Phonetic Transcription] was also the least used with only one instance observed in Google Assistant in Spanish, mirroring the numbers of Strategy 4 [Request Cancellation]. Strategy 9 [Instructions Provided] was likewise among the least employed strategies. It was utilized twice by Google, making up 6.67% of all Google strategies. Across languages, this strategy was used once in each, making up 3.33% of all strategies in each language, as well as 3.33% for all strategies used across both assistants. One notable omission from all the strategies collected is the use of the interactional “*huh?*” prevalent in HHI. Between the two languages tested, neither assistant produced “*huh?*” in any context.

Regarding the effect of assistant on the total number of strategies observed, Google used all 10 strategies, while Siri only used 7, leaving out strategies 4 [Request Cancellation], 9 [Instructions Provided], and 10 [Phonetic Transcription] which were exclusive to Google. A Bayesian test of association was run for the data from Task A. The resulting Bayes factor of 1.42:1 in favor of the alternative hypothesis indicated that there was some weak evidence for the non-independence of assistant and strategy occurrence within this task. The hypothesis for RQ3 centers on the observable difference in the frequency of repair strategy usage among the assistants. This difference is confirmed in this context. However, a more complete understanding of the correlation between these factors is gained by examining the combined data from both tasks, as discussed in Section 3.3.

The effect of language on the total number of strategies was likewise in question. English employed 90% of all strategies available, only excluding Strategy 10 [Phonetic Transcription]. Spanish similarly used 90% of all strategies, excluding Strategy 4 [Request Cancellation]. A test of association was performed, revealing a Bayes factor of 0.12:1 in favor of the null hypothesis, implying no link between language and strategy in this task alone. A full picture of the relation between language and strategy for both tasks is also captured in Section 3.3.

### 3.2 Task B- results

**FIGURE 3:**
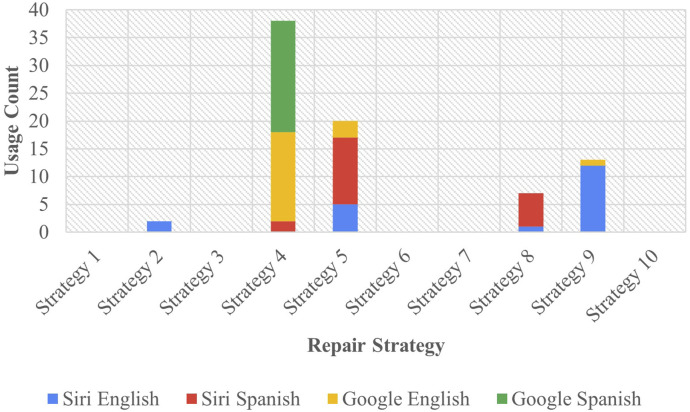
Task B results - assistant repair strategies.

In Task B, the researcher introduced the OIR initiator “*huh?*” (in English) or “*¿eh?*” (in Spanish) in dialogues with the assistant, noting their responses. The resultant data is summarized in Figure 3.

Strategy 4 [Request Cancellation] emerged as the most used approach in Task B, garnering 38 responses, which represented 47.5% of all strategies in this task. Google predominantly employed this strategy, constituting 90% of its total responses. Siri, on the other hand, only employed Strategy 4 [Request Cancellation] twice, comprising 5% of its overall output. This strategy represented 16 responses in English, making up 40% of all responses in that language, while Spanish utilized the strategy 22 times, accounting for 55% of all responses. Following this was Strategy 5 [Clarification Request], the second most utilized method, with 17 occurrences by Siri (42.5% of Siri’s output) and three for Google (7.5% of Google’s output), collectively amounting to 25% of all strategies employed. This strategy was utilized 12 times in Spanish (30% of all Spanish strategies) and 8 times in English (20% of all English strategies).

Regarding the strategies that appeared infrequently, Strategy 2 [Literal Interpretation]—the least used among the assistants—was used only twice by Siri (5% of the assistant’s output) and once in English, constituting 5% of all responses in English. In total, this strategy made up 2.5% of the overall responses. More significantly, many strategies were not utilized by any assistant in this task—strategies 1 [Partial Fulfillment], 3 [Misunderstanding], 6 [Internet Search], 7 [User Data Utilization], or 10 [Phonetic Transcription] were absent in both languages and across all assistants, marking a notable absence of 50% of all potential strategies at the assistants’ disposal.

The results demonstrated marked differences in strategy usage between Task A and Task B. In general, fewer strategies were used in Task B in response to the input introducing the reparative “*huh?*” in response to clarification requests from assistants. As noted, assistants in this task employed five strategies compared to Task A which included at least one instance of all 10 strategies. Task A predominantly utilized Strategy 2 [Literal Interpretation] and rarely utilized strategies 4 [Request Cancellation] or 10 [Phonetic Transcription], while Task B mainly employed the use of Strategy 4 [Request Cancellation], rarely used Strategy 2 [Literal Interpretation], and never used strategies 1 [Partial Fulfillment], 3 [Misunderstanding], 6 [Internet Search], 7 [User Data Utilization], or 10 [Phonetic Transcription].

A test of association was conducted between assistant and strategy for this task, revealing a Bayes factor of $2.54\times 10^{12}:1$ in favor of the alternative hypothesis. This indicates strong evidence supporting the non-independence between assistant and strategy. A secondary test of association was conducted to examine the relationship between language and strategy in this task, yielding a 1348:1 Bayes factor, affirming the notion of a relationship between language and strategy.

### 3.3 Task A and B- combined

A holistic examination of machine dialogue repair strategies was undertaken by combining the data from Task A and Task B. The key insight from the overall combined data was that Strategy 4 [Request Cancellation] emerged as the most frequently used, appearing 39 times, accounting for 27.86% of all strategies. Strategy 10 [Phonetic Transcription] was employed the least, occurring only once and making up only 0.71% of all strategies.

Concerning the utilization of strategies between assistants, Strategy 1 [Partial Fulfillment] was employed the least by Google, appearing only once, and accounting for 1.43% of all strategies used (and representing a total of 2.86% for the strategies utilized by both assistants combined). Conversely, the same assistant most frequently used Strategy 4 [Request Cancellation], which appeared 37 times, representing 52.86% of all its responses and 27.86% of all strategy usage between both assistants. The primary strategy employed by Siri was Strategy 5 [Clarification Request], used 20 times, making up 28.57% of its responses and 19.29% of all strategies in total. The least used strategy by Siri was Strategy 10 [Phonetic Transcription], which never employed it in either task—this strategy was only utilized as a response by Google Assistant, accounting for 0.71% of all strategy usage combined across both assistants.

Regarding the overall strategies used across languages, Strategy 4 [Request Cancellation] emerged as the predominant choice in English, constituting 24.29% of its total strategies used and 27.86% of all strategies combined. Strategy 10 [Phonetic Transcription] never appeared in English, with its sole instance in Spanish representing 0.71% of all strategies combined. In Spanish, Strategy 4 [Request Cancellation] was the most utilized, with this strategy appearing 22 times, making up 31.43% of its utilized strategies, out of a total of 27.86% across both assistants. Strategy 9 [Instructions Provided] saw the least use, appearing only once, accounting for 1.43% of total strategies used in Spanish and 10.71% of all strategies combined.

The analysis of task, language, and assistant, using data from both Task A and Task B combined revealed Bayes factors for each pair, demonstrating the relationships with strategy. Analysis revealed a significant relationship between the assistant and the strategy employed, with a Bayes factor of $1.69\times 10^{10}:1$ . Likewise, a notable correlation was observed for task and strategy, with a Bayes factor of $2.77\times 10^{16}:1$ . Language also displayed an association with a Bayes factor of 3.59:1 , although this proved to be the weakest relationship of the three factors.

### 3.4 Task C - results

In Task C, surveys were distributed in American English and Castilian Spanish to collect acceptability judgments on a five-point Likert scale from participants who were shown dialogues incorporating repair strategies used by the assistants from both Task A and Task B. To analyze these responses, this research utilized the cumulative link model package in R for ordinal logistic regression (Christensen, 2023)^7^. An ordinal logistic regression approach was chosen to take advantage of the inherent ordinal structure of the Likert scores, as treating ordinal data as continuous can introduce errors due to the assumption of normally distributed data with equal intervals between scale points—a concern discussed in Liddell and Kruschke (2018) and supported by Amidei et al. (2019) and Bürkner and Vuorre (2019). By capturing the relationships between strategy as a predictor and the Likert acceptability outcomes, this model allowed for an understanding of how different factors impacted the participants’ judgments. All code utilized in this analysis is provided in Galbraith (2024).

#### 3.4.1 English - task A

**FIGURE 4:**
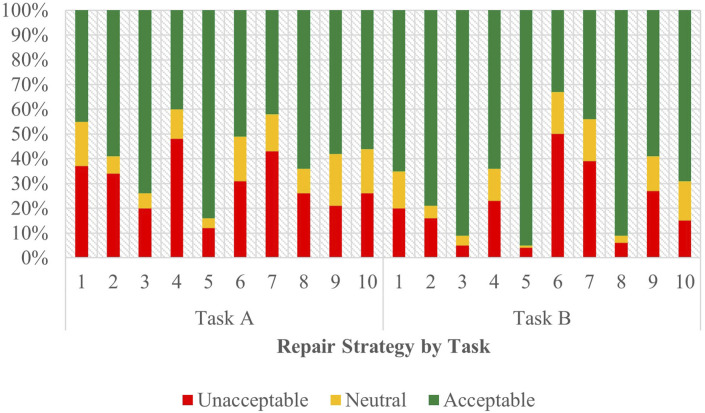
Task C acceptability judgment results - English task A and B.

As depicted in Figure 4, the acceptability judgements from the English survey are stratified by strategy and task, with Task A appearing on the left. The outcomes of the ordinal regression analysis performed on the data from Task A are presented in Supplementary Table 4 of the attachment.

From this data, Strategy 5 [Clarification Request] (coefficient = 1.85, SE = 0.27, *p* < .001), Strategy 3 [Misunderstanding] (coefficient = 1.23, SE = 0.26, *p* < .001) and Strategy 8 [App Suggestion] (coefficient = 0.62, SE = 0.25, *p* < 0.05) proved to have significant, positive effects when predicting user acceptability scores. The coefficients for strategies Strategy 2 [Literal Interpretation] (coefficient = 0.48, SE = 0.26, *p* = 0.06) and Strategy 10 [Phonetic Transcription] (coefficient = 0.47, SE = 0.25, *p* = 0.06) followed those of the three strategies listed above but lied outside the realm of significance by a slim margin. So while the coefficient order formed a pattern of preference, only the effects of Strategy 5 [Clarification Request], Strategy 3 [Misunderstanding], and Strategy 8 [App Suggestion] were significant enough to be conclusively ranked. Choice of assistant also demonstrated a significant influence (coefficient = 0.33, SE = 0.11, *p* < 0.01) on the acceptability judgments collected, underscoring the assistant’s role in shaping the participants’ evaluations.

#### 3.4.2 English - task B

Figure 4 displays the acceptability judgements from the English survey from Task B on the right. The outcomes of the ordinal regression analysis performed on the data from Task B are presented in Supplementary Table 5 of the attachment.

In this task, Strategy 5 [Clarification Request] (coefficient = 1.99, SE = 0.32, *p* < .001), Strategy 3 [Misunderstanding] (coefficient = 1.28, SE = 0.28, *p* < .001) and Strategy 8 [App Suggestion] (coefficient = 0.89, SE = 0.27, *p* < 0.01) all had significant, positive effects when predicting user acceptability scores. Conversely, Strategy 9 [Instructions Provided] (coefficient = −0.80, SE = 0.26, *p* < 0.01), Strategy 7 [User Data Utilization] (coefficient = −1.16, SE = 0.26, *p* < .001), and Strategy 6 [Internet Search] (coefficient = −1.61, SE = 0.26, *p* < .001) demonstrated increasingly negative scores. The use of assistant (coefficient = −0.14, SE = 0.12, *p* = 0.25) showed no significant effect on the perceived acceptability of the dialogues.

Compared to Task A, coefficient estimates for each strategy formed a more complete hierarchy of preference. Like Task A, strategies 5 [Clarification Request], 3 [Misunderstanding], and 8 [App Suggestion] (in descending order of their coefficients) topped the list as the most acceptable. Task B shows less uncertainty with only three strategies in regard to their significance—2 [Literal Interpretation], 10 [Phonetic Transcription], and 4 [Request Cancellation]. This data paints a more definitive picture of the acceptability ranking in this task, with strategies 9 [Instructions Provide], 7 [User Data Utilization], and 6 [Internet Search] being the least accepted among participants. As previously noted, and in contrast to Task A, the use of assistant in Task B exhibited no significant effect on acceptability.

#### 3.4.3 English - combined

**FIGURE 5:**
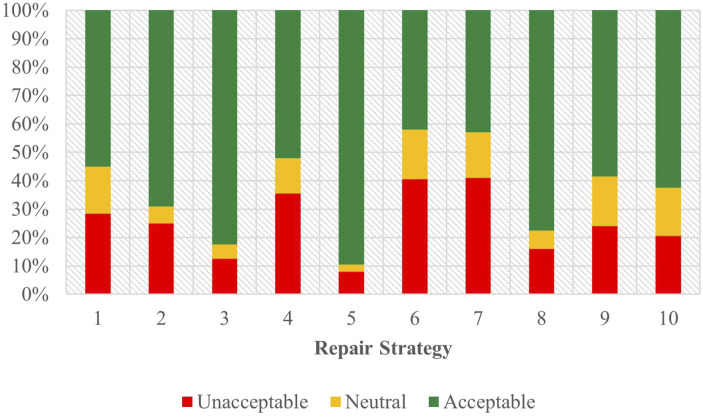
Task C acceptability judgment results - English combined.

Figure 5 shows the combined acceptability judgements for both Task A and Task B in English. The outcomes of the ordinal regression analysis performed on the data from the combined tasks are presented in Supplementary Table 6 of the attachment.

Here, Strategy 5 [Clarification Request] (coefficient = 1.87, SE = 0.20, *p* < .001), Strategy 3 [Misunderstanding] (coefficient = 1.24, SE = 0.19, *p* < .001), Strategy 8 [App Suggestion] (coefficient = 0.73, SE = 0.18, *p* < .001), and Strategy 2 [Literal Interpretation] (coefficient = 0.48, SE = 0.19, *p* < 0.05) all demonstrated significant, positive effects when predicting user acceptability scores. Strategy 4 [Request Cancellation] (coefficient = −0.36, SE = 0.18, *p* < 0.05), Strategy 7 [User Data Utilization] (coefficient = −0.56, SE = 0.18, *p* < 0.01) and Strategy 6 [Internet Search] (coefficient = −0.67, SE = 0.18, *p* < .001) displayed significant, negative effects, indicating increased odds of negative Likert scale responses. Data from strategies 9 [Instructions Provided] and 10 [Phonetic Transcription] suggested no conclusive influence on participant responses.

Beyond these strategies, the task performed emerged as a significant predictor (coefficient = 0.51, SE = 0.08, *p* < .001) that influenced the participants’ acceptability judgments, indicating an association between the nature of the task and how participants assessed the dialogues given. In contrast, the choice of assistant as a predictor (coefficient = 0.11, SE = 0.08, *p* = 0.18) did not demonstrate any significant impact on judgment scores in the combined English data.

#### 3.4.4 Spanish - task A

**FIGURE 6:**
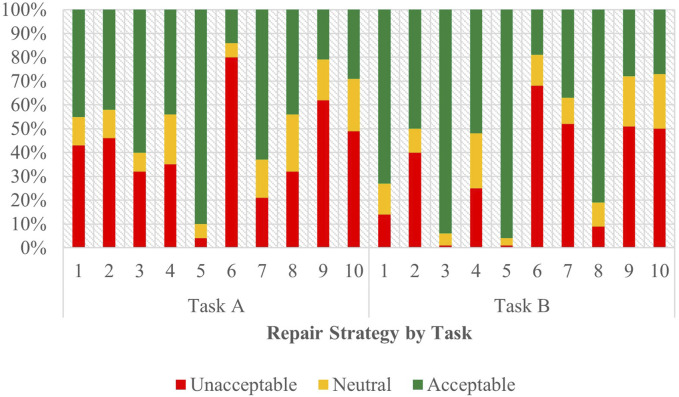
Task C acceptability judgment results - Spanish task A and B.

Task A results for the Spanish survey are visible on the left side of Figure 6, with full regression results in Supplementary Table 7 of the attachment.

Data from this task points to Strategy 5 [Clarification Request] (coefficient = 2.12, SE = 0.27, *p* < .001), Strategy 3 [Misunderstanding] (coefficient = 1.10, SE = 0.29, *p* < .001), and Strategy 7 [User Data Utilization] (coefficient = 0.77, SE = 0.25, *p* < .01) having a significant, positive effect on acceptability scores. Strategy 9 [Instructions Provided] (coefficient = −0.99, SE = 0.26, *p* < .001) and Strategy 6 [Internet Search] (coefficient = −1.61, SE = 0.26, *p* < .001), however, were significant indications of a negative score. Other than strategy, the choice of assistant demonstrated a measurable, influence (coefficient = 0.43, SE = 0.12, *p* < 0.001) on acceptability judgments in this task.

Comparing results from English Task A, strategies 5 [Clarification Request] and 3 [Misunderstanding] were also the most effective indications of producing a positive judgement. Strategy 7 [User Data Utilization], while among the top three significant predictors of positive scores in Spanish, was replaced by Strategy 8 [App Suggestion] in English, with Strategy 7 [User Data Utilization] being one of the strategies that produces the most negative sentiment in English (although this cannot be asserted with certainty due to its lack of significance). Strategies 6 [Internet Search] and 9 [Instructions Provided] in Spanish, which were indicative of negative scores, were likewise not sufficiently significant in Task A in English to make a firm determination of their standing—although from English’s coefficient ranking, they do not appear to be the least favored as they appear in the Spanish task. The effect of assistant was also present in both English and Spanish for Task A, with both languages demonstrating that assistant choice as a significant predictor of participant’ judgements.

#### 3.4.5 Spanish - task B

Task B results for the Spanish survey are depicted on the right side of Figure 6, with full regression results in Supplementary Table 8 of the attachment.

Results indicated that Strategy 5 [Clarification Request] (coefficient = 2.24, SE = 0.32, *p* < .001) and Strategy 3 [Misunderstanding] (coefficient = 1.67, SE = 0.28, *p* < .001) had significant, positive effects when predicting user acceptability scores. Strategy 4 [Request Cancellation] (coefficient = −0.63, SE = 0.26, *p* < .05), Strategy 2 [Literal Interpretation] (coefficient = −0.92, SE = 0.26, *p* < .001), Strategy 7 [User Data Utilization] (coefficient = −1.65, SE = 0.26, *p* < .001), Strategy 10 [Phonetic Transcription] (coefficient = −1.72, SE = 0.26, *p* < .001), Strategy 9 [Instructions Provided] (coefficient = −1.75, SE = 0.25, *p* < .001), and Strategy 6 [Internet Search] (coefficient = −2.22, SE = 0.26, *p* < .001) were all correlated with increasingly negative scores. Choice of assistant (coefficient = 0.45, SE = 0.12, *p* < .001) was additionally seen to have an impact in Task B. The sole predictor which had an uncertain effect on user score was Strategy 8 [App Suggestion] (coefficient = 0.11, SE = 0.25, *p* = 0.67) which, when compared to the baseline, did not demonstrate a substantial impact.

Comparing Task B to Task A revealed variations in the predicting factors in Spanish. Strategy 7 [User Data Utilization] in Task A had a positive effect on user scores, while demonstrating a negative effect in Task B. And while in Task A the contribution of strategies 4 [Request Cancellation], 2 [Literal Interpretation], and 10 [Phonetic Transcription] towards user scores were uncertain, their coefficients suggested that the ranking of these strategies may differ compared to Task B. Despite this, strategies 3 [Misunderstanding] and 5 [Clarification Request] continue to emerge as the top predictors with a positive effect, with strategies 9 [Instructions Provided] and 6 [Internet Search] being the predictors with the least positive effect in across both tasks.

#### 3.4.6 Spanish - combined

**FIGURE 7:**
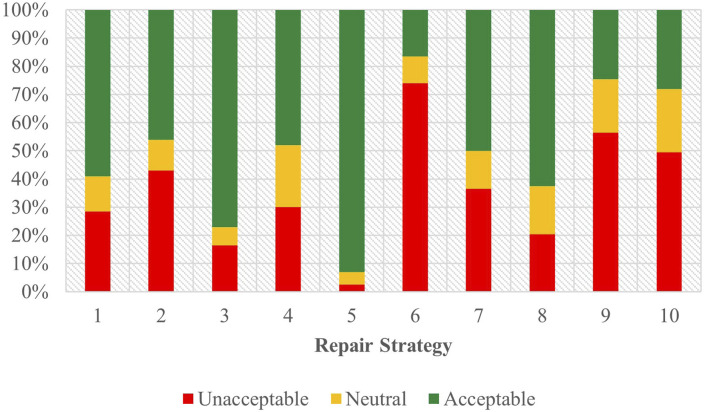
Task C acceptability judgment results - Spanish combined.

Figure 7 shows the combined acceptability judgements for both Task A and Task B in Spanish, with the full regression results visible in Supplementary Table 9 of the attachment.

In this combined data Strategy 5 [Clarification Request] (coefficient = 2.08, SE = 0.20, *p* < .001) and Strategy 3 [Misunderstanding] (coefficient = 1.38, SE = 0.20, *p* < .001) emerged as the most influential strategies that were significantly associated with higher participant judgements. In contrast, Strategy 2 [Literal Interpretation] (coefficient = −0.50, SE = 0.18, *p* < .01), Strategy 10 [Phonetic Transcription] (coefficient = −1.04, SE = 0.18, *p* < .001), Strategy 9 [Instructions Provided] (coefficient = −1.34, SE = 0.18, *p* < .001), and Strategy 6 [Internet Search] (coefficient = −1.87, SE = 0.18, *p* < .001) were more strongly correlated with negative user judgments. Strategies 8 [App Suggestion], 4 [Request Cancellation], and 7 [User Data Utilization] showed no significant effects when compared to the baseline for both tasks. Regarding the predicting factors beyond strategy, assistant choice (coefficient = 0.41, SE = 0.08, *p* < .001) produced a markedly positive effect on participant scores.

Comparing these findings with the combined task data for English revealed differences between the predictor and acceptability score outcomes for each language. In both English and Spanish, Strategies 5 [Clarification Request] and 3 [Misunderstanding] emerged as the top two predictors for positive acceptability scores from users. Among the strategies used the least, Strategy 6 [Internet Search] was consistently regarded as the least favorable strategy across languages. Notable differences could be seen regarding Strategy 2 [Literal Interpretation], which was associated with a higher acceptability score in English, but a more negative score in Spanish, although both effects were relatively weak compared to the baseline strategy tested. Additionally, Strategy 4 [Request Cancellation] and Strategy 7 [User Data Utilization] in English had significant associations with a negative acceptability, whereas in Spanish these strategies, although likewise having negative coefficients, did not rise to a level of significance. The Spanish data also indicated that both task and assistant significantly predicted user scores, whereas in English, only the task variable showed such predictive power.

#### 3.4.7 English and Spanish - combined

**FIGURE 8:**
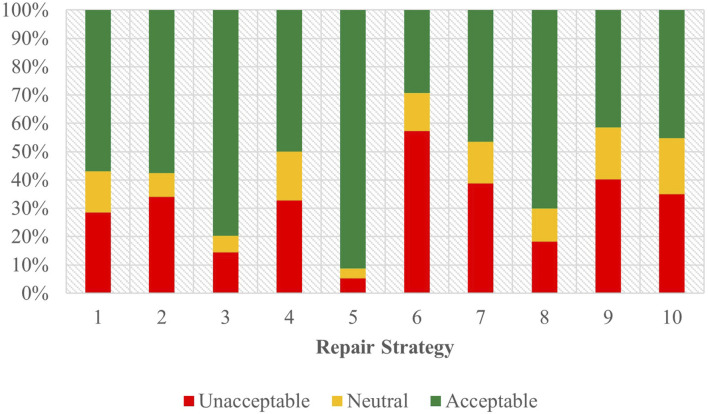
Task C acceptability judgment results - English and Spanish combined.

**FIGURE 9:**
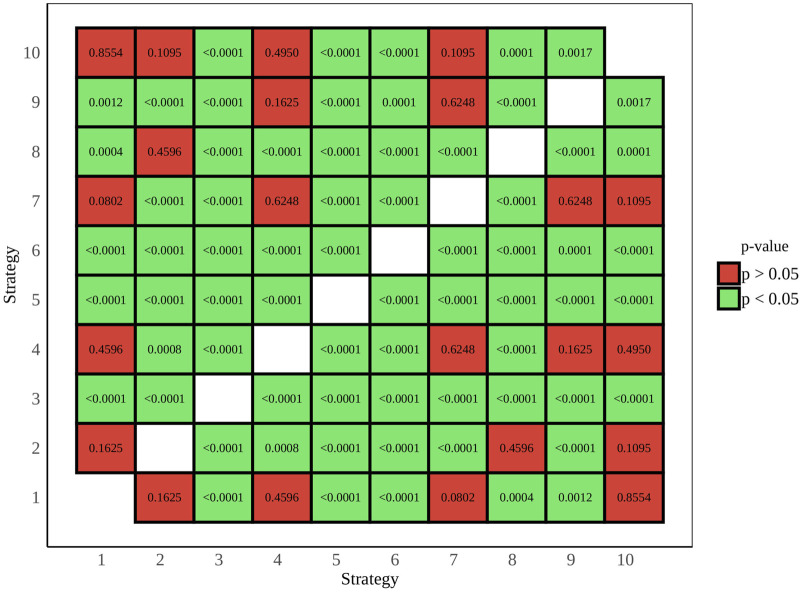
Task C *p*-value pairwise comparison - English and Spanish combined.

**FIGURE 10:**
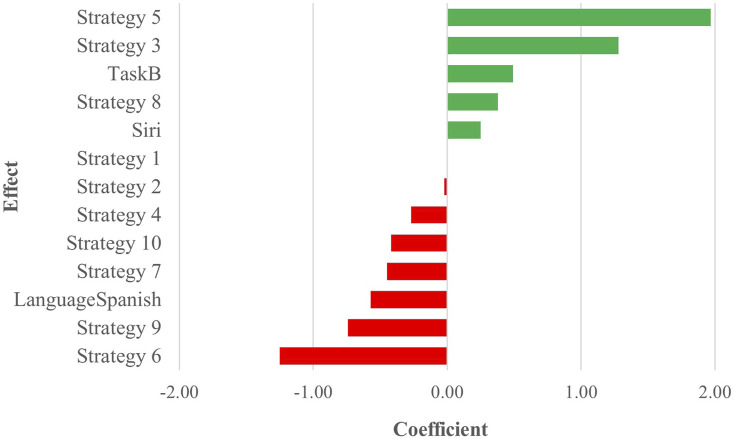
Task C ranked regression coefficients - English and Spanish combined.

All acceptability judgments gathered from participants across both tasks and languages are depicted in Figure 8, offering a comprehensive overview of participant responses. *p*-value pairwise comparisons between individual strategies are provided in Figure 9, while a full view of regression data is available in Supplementary Table 10 of the attachment. An overview of all coefficient rankings in this combined data is displayed in Figure 10.

Within this combined dataset, all strategies, except for Strategy 2 [Literal Interpretation] (coefficient = −0.02, SE = 0.13, *p* = 0.87), demonstrated significant effects on participant scores. Strategy 5 [Clarification Request] (coefficient = 1.97, SE = 0.14, *p* < .001) emerged as the most influential indicator of a positive acceptability score, followed by Strategy 3 [Misunderstanding] (coefficient = 1.28, SE = 0.14, *p* < .001), which exhibited a similar, though slightly weaker, positive effect. Additionally, Strategy 8 [App Suggestion] showed a noteworthy association with positive Likert scores (coefficient = 0.38, SE = 0.13, *p* < .01). Conversely, Strategy 4 [Request Cancellation] (coefficient = −0.27, SE = 0.13, *p* < .05), Strategy 10 [Phonetic Transcription] (coefficient = −0.42, SE = 0.13, *p* < .01), Strategy 7 [User Data Utilization] (coefficient = −0.45, SE = 0.13, *p* < .001), Strategy 9 [Instructions Provided] (coefficient = −0.74, SE = 0.13, *p* < .001), and Strategy 6 [Internet Search] (coefficient = −1.25, SE = 0.13, *p* < .001) were all linked to increasingly lower acceptability judgments, with Strategy 6 [Internet Search] displaying the most pronounced degree of unfavorability.

Apart from the variables of strategies, assistant, task, and language also demonstrated significant effects on user scores. Comparing the two tasks, the choice of Task B (coefficient = 0.49, SE = 0.06, *p* < .001) was found to have a notable impact compared to Task A. Similarly, a change in assistant (coefficient = 0.25, SE = 0.06, *p* < .001) was identified as a significant predictor of user scores when comparing Siri to the baseline, Google Assistant. Language choice (coefficient = −0.57, SE = 0.06, *p* < .001) also emerged as a significant factor, suggesting a negative association when comparing Spanish to English as a baseline, thus indicating comparatively more negative scores.

## 4 Discussion

Data from Task A, designed to elicit a response like the repair initiator “*huh?*”, demonstrates that assistants do not produce “*huh?*” in either English or in Spanish, affirming the hypothesis for RQ2. As predicted, assistants avoid the use of this interactional repair initiator, opting for a variety of different repair methods. Contrasting this with dialogue repair in HHI, it could be inferred that the avoidance of *unspecified* repair stems from its potential inefficiency. That is, *unspecified* repair often results in repeated misunderstandings, prolonging the dialogue. This differs from *interrogative* repair in which the speaker uses an interrogative to specify the source of the miscommunication in the prior turn. As such, HCI appears to keep interaction to a minimum by avoiding extraneous dialogue turns. This is possibly reflective of the task-oriented nature of the assistants, with dialogues containing fewer turns being favored to expediate the resolution of the user’s query.

Not only do the assistants lack the capacity to employ “*huh?*” but they also struggle to comprehend it, thus addressing RQ1 which questioned how assistants would manage dialogue repair involving “*huh?*”. This pattern appears in Task B results, where assistants addressed the use of “*huh?*”. Siri mainly uses Strategy 5 [Clarification Request] which asks for the appropriate information as a repair strategy (the preferred choice of users) and shows a variety of responses overall, employing half of all possible strategies recorded in the present investigation. Conversely, Google Assistant mainly uses Strategy 4 [Request Cancellation] which expresses an inability to perform the task (a strategy that is among the least favored strategies by users) and produces fewer strategies overall. In this sense, RQ1’s hypothesis was partially confirmed, as it suggested that the assistants would face difficulties in accurately interpreting and managing instances of “*huh?*”. While Siri’s strategy use more closely aligns with user preference than Google Assistant in this task, it still only utilizes a limited number of strategies compared to Task A. Comparatively, Google Assistant’s response is notably sub-par in that it only utilizes a few possible strategies, many of which are not strategies preferred by users. This results in outcomes reminiscent of Stommel et al. (2022) criticisms of interactions with robots as “rigid” and “arguably asocial”. The differences in Siri and Google Assistant’s responses to the introduction of “*huh?*” in Task B underscore the significant impact of neglecting interactional repair in the development of assistants.

Results from Task B also shed light on RQ3, which hypothesized that there would be a variation in the quality and frequency of repair strategies used among the languages examined, with Spanish assistants performing poorer due to the disparity in resources allocated to Spanish language development. While results demonstrate an expected difference in strategy usage frequency between English and Spanish, Spanish consistently uses the top three most preferable strategies in both tasks. In this regard, Spanish yields a higher quality outcome by employing the most favored strategies. These findings suggest the presence of a potential difference in the underlying mechanisms governing dialogue repair strategies between languages.

Acceptability scores from Task C among participants revealed a discernible hierarchy of preference for repair strategies—when combined, the English and Spanish data indicate a preference for asking for appropriate information (Strategy 5 [Clarification Request]), while disliking when the assistant searches the internet for answers (Strategy 6 [Internet Search]). This aversion towards Strategy 6 [Internet Search] could stem from its failure to directly address the need for repair in the dialogue, unlike Strategy 5 [Clarification Request]. This acknowledgement is illustrated in the second step of the speaker paradigm:

Speaker1:repairrequest→ Speaker2:acknowledgementofrepairrequest →Speaker2:solutiontorepairmisunderstanding.

While Conversational Analysis suggests that the progressive movement of the dialogue itself can constitute a successful repair in HHI, the framework also explains that the response to the repair is typically made evident from the resolution in the subsequent dialogue turn (Albert and de Ruiter, 2018). Users’ preferred strategies, Strategy 5 [Clarification Request] and Strategy 3 [Misunderstanding], appear to display this behavior of addressing the source of the miscommunication from the previous turn. Strategy 8 [App Suggestion], however, takes a different approach by not directly verbally acknowledging the repair request. Instead, it employs a kind of multimodal method by opening an application relevant to the request. This could be likened to a physical gesture in HHI, which an interlocutor could produce to acknowledge the other speaker’s repair request during an interaction. In Strategy 8 [App Suggestion] the virtual assistant substitutes this physical gesture with a “gestural” opening of the application, effectively addressing the source of the miscommunication from the previous turn through this “gesture”. This explanation could hold implications for the field of HRI, considering that anthropomorphic robots possess a physical gestural capability that mirrors this interactional component.

Strategy 6 [Internet Search], the least favored strategy, exhibited an indirect form of acknowledgment of the need for repair and ultimately provided an unsatisfactory resolution to the user’s query. This strategy attempted to search the internet, presenting an ostensibly similar acknowledgement to Strategy 8 [App Suggestion], by opening the browser to initiate a search for the required information. This approach proves to be less productive compared to Strategy 8 [App Suggestion], however, which opens the application relevant to the parsed intent behind the user query. That is, if the user requests the assistant to “*play the new album of* {*unintelligible phrase*}”, Strategy 8 [App Suggestion] opens the YouTube application, therefore displaying a more nuanced understanding of the intent behind the user’s request as opposed to simply opening the browser. This deeper understanding of the query could explain its higher acceptability among participants. Likewise, Strategy 6 [Internet Search]’s approach also appears to shift the burden to the user by requiring them to take an additional step to locate the necessary information on the internet. This broader request contrasts with the approach of Strategy 8 [App Suggestion], which narrows the search to a specific application.

The remaining least-preferred strategies likewise exhibited ambiguous acknowledgment of the source of the miscommunication. A notable finding from the examination of these strategies was the rejection of the hypothesis outlined in RQ4, which suggested that users would strongly favor strategies focused on attempting to fulfill requests at any cost. The analysis revealed that even a strategy like Strategy 10 [Phonetic Transcription], which failed to provide a satisfactory acknowledgment and did not offer a resolution to the user’s query, proved to be more favorable than Strategy 6 [Internet Search]. Other strategies similarly did not address the source of miscommunication, such as Strategy 7 [User Data Utilization] which utilized user information to fulfill a request while disregarding any recognition of a misunderstanding, resulting in an erroneous outcome. Strategy 9 [Instructions Provided] sidestepped addressing the source of the error, opting to provide the user with instructions for using the assistant (reminiscent of the open-ended gesture seen in Strategy 6 [Internet Search], which proved more burdensome than beneficial for the user). Strategy 4 [Request Cancellation] indirectly addressed the source of miscommunication by acknowledging the assistant’s inability to fulfill the request, an action which recognized that an error had occurred but did not inform the user of its source. When Strategy 4 [Request Cancellation] remained completely silent to convey its inability to process the request, it more clearly followed the pattern of not directly addressing the source of miscommunication. This suggests the possible need to divide Strategy 4 [Request Cancellation] into two distinct strategies in future analyses, with the aim of exploring each individually to develop a more comprehensive understanding of their respective impacts on user evaluations. Additionally, there is a need to examine any correlation between strategies that implicitly acknowledge the source of repair, yet still exhibit poor user acceptability—this might suggest that, despite ambiguously acknowledging the miscommunication’s source, the lack of a satisfactory query resolution might significantly influence user preferences. This could shed light on the underlying factors contributing to the variability in the impact of repair strategies that were found to be closely linked with negative outcomes in this study.

Overall, there is a notable mismatch between the repair strategies utilized by Google Assistant and Siri and user preferences. The only positive concordance between user preference and machine use is observed for the least utilized and least acceptable strategy in Task B. However, this alignment may be coincidental, as Task B generally employed fewer strategies, therefore incidentally coinciding with the least acceptable strategy matching user preference in this task. These discrepancies underscore the need for further research to enhance the capabilities of CUIs, not only in virtual assistants but also in interactive robotic systems embodying these assistants and their limitations in a human-like form. This research should aim to develop and implement repair strategies that more closely align with human interactional language, while also considering user feedback from languages including English, Spanish, and beyond.

## 5 Limitations

### 5.1 Metrics beyond acceptability judgements

Despite the reliability and robustness of acceptability judgments, as described in Section 2.2.1, there remains a question of whether a single judgment from users can entirely capture the full extent of a dialogue’s appropriateness in a given context. This question opens avenues for future projects to investigate systems that gather a broader array of data from sources beyond one-off acceptability judgments. Such avenues may include CUIs that integrate reinforcement learning based on immediate human feedback. Utilizing an assistant capable of self-training based on real-time user judgements would offer a more comprehensive approach to dialogue management by empowering users to customize the assistant to their preferences, while also compiling a larger pool of judgment data. This approach could have significant impact on interactions in HRI, whereby researchers could create robots that modify their physical behavior in response to acceptability judgements informed by reinforcement feedback, possibly producing more interactive and satisfactory interactions in scenarios unrestricted by voice-only communication.

### 5.2 CUI variability

The dynamic nature of CUIs, particularly those that are closed-source like the systems tested in this study, presents a challenge due to their dialogue managers which are constantly evolving and inaccessible for observation. The underlying operations of these systems, hidden from view, are subject to change without explicit explanation from developers. Moreover, the systems under study—Apple’s Siri and Google’s Google Assistant—are currently undergoing a substantial shift in their back-end operations as they transition to systems powered by large language models (LLMs). LLMs depart significantly from form-filling techniques, functioning as open-domain systems employing probability algorithms to generate responses rather than a series of fixed intents. Despite these advancements, the investigation of user preferences remains relevant for assessing the output of these emerging systems and for future studies focused on evaluating the acceptability of responses from next-generation CUIs.

### 5.3 Repair strategy categorization

Another limitation concerns the categorization of the repair strategies. Although the current study sought to categorize assistant responses into general strategies with similar effects and provide a succinct overview of system behavior, there remains a necessity for more in-depth exploration to document the full range of potential strategies. This is particularly crucial given the constantly evolving nature of CUIs, which demands an up-to-date documentation of their outputs along with any variations in user interaction patterns. As discussed in Section 2.2.2, the classification framework used in this study was designed to categorize repair strategies based on the minimal actions an assistant could take to address a dialogue breakdown, with consideration of whether these actions effectively furthered the conversation or resulted in significant interruptions. The categorization utilized in this experiment also took into account and expanded upon HHI repair strategies found in Schegloff et al. (1977), while drawing inspiration from the framework established by Motta and Quaresma (2022) which consisted of eight categories of repair. While these categories laid a solid foundation for further exploration, some encompassed multiple strategies used in the present study. This highlighted a difference in methodological approach, as the present investigation endeavored to deconstruct those groups into bespoke categories through the documentation of the behaviors demonstrated by the assistants. Future research would benefit from a focus on refining the categories of repair strategies utilized by current assistants, with a particular emphasis on documenting any emergent repair strategies as CUIs continue to evolve. Additionally, as human interactions with robots progresses and becomes more sophisticated, research could extend to encompass the categorization of HRI-specific repair strategies, allowing for the implementation of more effective mechanisms for handling errors and misunderstandings. This would ultimately improve the user experience, guaranteeing that interactions align with users’ preferences and expectations, while also enhancing the performance and reliability of robots in real-world scenarios.

### 5.4 Text-based feedback from assistants

One limitation of this study concerns the lack of detailed documentation regarding the textual output displayed by the voice assistants’ applications during testing. While the research primarily focused on voice-based interactions, occasional notations of text outputs were made but were not systematically recorded. This was partly due to the nature of how the assistants often processed requests without providing explanatory text, leading to varied categorizations within Table 1. An exception to this pattern includes Strategy 6 [Internet Search], Strategy 8 [App Suggestion], and Strategy 10 [Phonetic Transcription], where the system provided textual or visual feedback, influencing their categorization as distinct repair strategies. While recording this text-based output could have enhanced the depth of the analysis, it would have been applicable only to specific strategies, as many did not exhibit distinctive text outputs that would have altered their categorization. Nevertheless, the absence of recorded text outputs for strategies using this modality constitutes a limitation of this study.

## Data Availability

The original contributions presented in the study are included in the article/Supplementary Material, further inquiries can be directed to the corresponding author.

## Footnotes

1 iOS does not provide explicit version information for individual system applications like Siri.

2 Both languages share the same wake word for Google Assistant.

3 The queries performed in Task A are provided in Supplementary Table 1 of the attachment.

4 The queries performed in Task B are provided in Supplementary Table 2 of the attachment.

5 The decision to utilize 20 queries instead of 30 (as performed in Task A) was motivated by the multi-turn nature of this task which effectively provides more than 20 individual data points - “*Researcher* → *Assistant* → *Researcher* → *Assistant* → *Researcher* → *Assistant*.”

6 To ensure consistency, the dialogue presented to the user followed a similar structure and repair strategy as a template. This was done in cases where a specific strategy was not employed by an assistant in one of the languages or task categories, necessitating the creation of a dialogue that replicated the observed strategy.

7 This model used a baseline of Strategy 1 [Partial Fulfillment], Google Assistant, and English as a basis of comparison for the analysis.

## Supplementary material

SUPPLEMENTARY TABLE S1 Task A Utterances with Strategy Labels.

•**Request - User**: Queries asked of both assistants in English and Spanish by the researcher. The introduction of the unintelligible phrase for each language is noted in curly brackets.
•**Response - Assistant**: Assistants’ responses derived from the queries in **Request - User**. Any non-verbal actions performed by assistants are noted between brackets.
•**Machine Repair Strategy**: Repair strategy labels for the responses in **Response - Assistant**.

SUPPLEMENTARY TABLE S2 Task B Utterances with Strategy Labels.

•**Request - User**: Queries asked of both assistants in English and Spanish by the researcher.
•**Response 1 - Assistant**: Assistants’ responses derived from the queries in **Request–User**. Any non-verbal actions performed by assistants are noted between brackets.
•**Repair Initiator–User**: The introduction of the OIR initiator *“huh?”*.
•**Response 2 and 3–Assistant**: Assistants’ responses derived from the introduction of the OIR in **Repair Initiator–User**. This includes two responses as the assistant first responds to *“huh?”*, then the same OIR initiator is introduced again in order to elicit a second response from the assistant which would possibly contain a different repair strategy. These responses are separated by a forward slash. Any non-verbal actions performed by assistants are noted between brackets.
•**Machine Repair Strategy 1**: Repair strategy labels for the responses to the first introduction of the OIR initiator *“huh?”* in **Response 2 and 3–Assistant**.
•**Machine Repair Strategy 2**: Repair strategy labels for the responses to the second introduction of the OIR initiator *“huh?”* in **Response 2 and 3–Assistant**.

SUPPLEMENTARY TABLE S3 Task C Utterances with Strategy Labels.

•**Sample Dialogues–Task A–*English / Spanish***
   - **Strategy**: The numbered strategy (derived from Table 1) from Task A in either English or Spanish with two versions, A and B, which with A representing Siri and B representing Google Assistant (that is, 1A and 1B represent Strategy 1 from A–Siri, and Strategy 1 from B–Google Assistant). This data was compiled in an attempt to get aggregate examples of a single strategy from both assistants to display to participants in the survey in Task C.
 - **Utterance**: A set of dialogues presented to users in Task C’s survey, reflective of the corresponding repair strategy listed in **Strategy** for Task A.
•**Sample Dialogues–Task B–*English / Spanish***
   - **Strategy**: The numbered strategy (derived from Table 1) from Task B in either English or Spanish with two versions, A and B, which with A representing Siri and B representing Google Assistant (that is, 1A and 1B represent Strategy 1 from A–Siri, and Strategy 1 from B–Google Assistant). This data was compiled in an attempt to get aggregate examples of a single strategy from both assistants to display to participants in the survey in Task C.
 - **Utterance**: A set of dialogues presented to users in Task C’s survey, reflective of the corresponding repair strategy listed in **Strategy** for Task B.

SUPPLEMENTARY TABLES S4–S10 Task C Ordinal Regression Data.
Supplementary Tables 4 through 10 display ordinal regression results from using the ordinal package in R when comparing the participant acceptability judgments with possible predicting factors from the study including strategy, language, task, and assistant—as seen in the “Effect” column of each table. The lower half of the final table, Supplementary Table 10, has *p*-value pairwise comparisons between strategies for the combined English and Spanish data.
