# Supplementary material for: An analysis of dialogue repair in virtual assistants
Source: Front Robot AI. 2024 Nov 11;11:1356847. doi: 10.3389/frobt.2024.1356847 (PMC11586770; doi:10.3389/frobt.2024.1356847)
Supplement: Supplementary file 1 [file DataSheet1.pdf]

Supplementary Table 1. Task A Utterances with Strategy Labels

Task A Siri English		
**Request - User**	**Response - Assistant**	**Machine Repair Strategy**
I have an appointment at the dentist for *{unintelligible phrase}*.	There's nothing scheduled at [user's home address]	7
How can I get to the street *{unintelligible phrase}*?	[Opens map app]	8
Play the song *{unintelligible phrase}*.	[Opens music app]	8
{unintelligible phrase}.	Good afternoon.	3
Are there any *{unintelligible phrase}* in Barcelona?	[Opens maps, searches "Wawa" after attempt to transcribe]	2
How long does it take from Balmes street to *{unintelligible phrase}*?	[Opens map app]	8
Call *{unintelligible phrase}*.	[Attempts to call contact "blumenbox" after attempt to transcribe]	2
Send a message to *{unintelligible phrase}*.	[Attempts to send message to "my wife" after attempt to transcribe]	2
What's the forecast for today in *{unintelligible phrase}*.	[Looks for forecast in Ecuador after attempt to transcribe]	6
Where's a *{unintelligible phrase}* nearby?	What kind of businesses are you looking for?	5
Play the new album of *{unintelligible phrase}*.	[Plays "new music" playlist from music app]	2
Send a text message to Maria that says: *{unintelligible phrase}*.	[Attempts to send message "all alarms" after attempt to transcribe]	2
Create a reminder for *{unintelligible phrase}*.	[Prompts the user to download an app for reminders]	1
What is the capital of *{unintelligible phrase}*?	[Looks up the capital of the USA from internet]	6

How long is the *{unintelligible phrase}*?	[Opens alarms app after attempt to transcribe "all alarms"]	2
**Task A Siri Spanish**		
**Request - User**	**Response - Assistant**	**Machine Repair Strategy**
Tengo una cita en el dentista para *{unintelligible phrase}*.	No tienes nada en el dentista en tu calendario.	1
¿Cómo puedo llegar a la calle *{unintelligible phrase}*?	[Opens maps app]	8
Pon la canción *{unintelligible phrase}*.	[Opens music app]	8
{unintelligible phrase}	¡Hola!	3
¿Hay algún *{unintelligible phrase}* en Barcelona?	[Tells the temperature in Barcelona]	1
¿Cuánto se tarda desde la calle Balmes a *{unintelligible phrase}*?	[Looks up "calle Balmes" on map app]	2
Llama a *{unintelligible phrase}*.	¿A quién?	5
Envía un mensaje a *{unintelligible phrase}*.	[Attempts to send a message "Dojo" after attempt to transcribe]	2
¿Cuál es el pronóstico de hoy en *{unintelligible phrase}*?	¿A quién?	5
¿Dónde hay una *{unintelligible phrase}* cerca?	[Looks up "bars" on internet after attempt to transcribe]	6
Reproduce el nuevo álbum de *{unintelligible phrase}*.	[Plays latest album by ABBA on music app after attempt to transcribe]	2
Envía un mensaje de texto a María que diga: *{unintelligible phrase}*.	[Attempts to send message "Hola" to María after attempt to transcribe]	2
Crea un recordatorio para *{unintelligible phrase}*.	No veo ninguna app para eso. Tendrás que descargarte una.	8

¿Cuál es la capital de *{unintelligible phrase}*?	[Looks up capital of "Barbacoa" on internet after attempt to transcribe]	6
¿Cuánto mide el *{unintelligible phrase}*?	[Looks up "size of dogs" on internet after attempt to transcribe]	6
**Task A Google English**		
**Request - User**	**Response - Assistant**	**Machine Repair Strategy**
Set an appointment for the dentist at *{unintelligible phrase}*.	Got it, when is the event? [Opens dialogue for event entitled "Dentist at"]	2
How can I get to the street *{unintelligible phrase}*?	The best way to get to [user's home address] is…	7
Play the song *{unintelligible phrase}*.	[Opens YouTube app]	8
{unintelligible phrase}.	[Gives instructions about possible actions with the assistant]	9
Are there any *{unintelligible phrase}* in Barcelona?	[Looks up "Barcelona" on internet]	6
How long does it take from Balmes street to *{unintelligible phrase}*?	[Looks up "Balmes Street" on internet]	6
Call *{unintelligible phrase}*.	Who do you want to call? [Presents list of contacts]	5
Send a message to *{unintelligible phrase}*.	Who do you want to message? [Presents list of contacts]	5
What's the forecast for today in *{unintelligible phrase}*?	[Gives forecast for user's current location]	7
Where's a *{unintelligible phrase}* nearby?	[Gives information about user's current location]	7
Play the new album of *{unintelligible phrase}*.	I looked for that, but it either isn't available or can't be played right now.	4
Send a text message to Adrian and Sofia that says the following: *{unintelligible phrase}*.	Looks like there's no phone number for Adrian.	1

Create a reminder for *{unintelligible phrase}*.	What's the reminder?	5
What is the capital of *{unintelligible phrase}*?	[Looks up the "capital of Alabama" on internet after attempt to transcribe]	6
How long is the *{unintelligible phrase}*?	How long is what?	5
**Task A Google Spanish**		
**Request - User**	**Response - Assistant**	**Machine Repair Strategy**
Apunta que tengo una cita en el dentista para *{unintelligible phrase}*.	He guardado tu nota [creates note that transcribes “lalala”].	2
¿Cómo puedo llegar a la calle *{unintelligible phrase}*?	[Opens map app and searches for directions to user’s home address]	2
Pon la canción *{unintelligible phrase}*.	[Opens YouTube app]	8
{unintelligible phrase}	[No response, unintelligible phrase partially parsed]	10
¿Hay algún *{unintelligible phrase}* en Barcelona?	[Searches for "tata" on browser after attempt to transcribe]	6
¿Cuánto se tarda desde la calle Balmes a *{unintelligible phrase}*?	[Searches in map application directions from user’s home address to Balmes street]	7
Llama a *{unintelligible phrase}*.	[Searches for contacts after attempt to transcribe]	2
Envía un mensaje a *{unintelligible phrase}*.	Lo siento, no te he entendido.	3
¿Cuál es el pronóstico de hoy en *{unintelligible phrase}*?	[Gives user’s current location forecast]	7
¿Dónde hay una *{unintelligible phrase}* cerca?	[Gives instructions about possible actions with the assistant]	9
Reproduce el nuevo álbum de *{unintelligible phrase}*.	[Opens YouTube app]	8

Envía un mensaje de texto a Adrian y Sofia que diga lo siguiente: *{unintelligible phrase}*.	Lo siento, no te he entendido.	3
Crea un recordatorio para *{unintelligible phrase}*.	Lo siento, no te he entendido.	3
¿Cuál es la capital de *{unintelligible phrase}*?	[Looks up the "capital" on internet]	6
¿Cuánto mide el *{unintelligible phrase}*?	[Looks up the phrase ¿cuánto mide el…? on internet]	6

Supplementary Table 2. Task B Utterances with Strategy Labels

**Task B Siri English**					
**Request - User**	**Response 1 - Assistant**	**Repair Initiator - User**	**Response 2 & 3 – Assistant**	**Machine Repair Strategy 1**	**Machine Repair Strategy 2**
Set an alarm.	For what time?	huh?	For what time? / For what time?	5	5
Set a reminder.	You'll need an app for that	huh?	You'll need an app for that / Siri's here to help. Get to know Siri at apple.com/siri.	8	9
Set a timer.	For how long?	huh?	For how long? / For how long?	5	5
Add something to my calendar.	What do you want to call it?	huh?	When should I schedule it for? / Siri's here to help. Get to know Siri at apple.com/siri.	5	9
Send a text message.	Who do you want to message?	huh?	I don't see "huh" in your contacts. / I don't see "huh" in your contacts.	2	2
Add to my shopping list.	I don't see an app for that.	huh?	Siri's here to help. Get to know Siri at apple.com/siri. / Siri's here to help. Get to know Siri at apple.com/siri.	9	9
Make a call.	To who?	huh?	Siri's here to help. Get to know Siri at apple.com/siri. / Siri's here to help. Get to know Siri at apple.com/siri.	9	9

Start a video chat.	To who?	huh?	Siri's here to help. Get to know Siri at apple.com/siri. / Siri's here to help. Get to know Siri at apple.com/siri.	9	9
Start a list.	Sorry I can't help with that.	huh?	Siri's here to help. Get to know Siri at apple.com/siri. / Siri's here to help. Get to know Siri at apple.com/siri.	9	9
Send an email	Doesn't look like you have an app to do that.	huh?	Siri's here to help. Get to know Siri at apple.com/siri. / Siri's here to help. Get to know Siri at apple.com/siri.	9	9
**Task 2 Siri Spanish**					
**Request - User**	**Response 1 - Assistant**	**Repair Initiator - User**	**Response 2 & 3 – Assistant**	**Machine Repair Strategy 1**	**Machine Repair Strategy 2**
Pon una alarma.	¿Para cuándo configuro la alarma?	¿eh?	¿Para cuándo configuro la alarma? / ¿Para cuándo configuro la alarma?	5	5
Configura un recordatorio.	No veo ninguna app para eso. Tendrás que descargarte una.	¿eh?	No veo ninguna app para eso. Tendrás que descargarte una. / No veo ninguna app para eso. Tendrás que descargarte una.	8	8
Pon un temporizador.	¿De cuánto tiempo?	¿eh?	¿De cuánto tiempo? / ¿De cuánto tiempo?	5	5
Crea un evento de calendario.	¿Qué nombre quieres ponerle?	¿eh?	¿Qué nombre quieres ponerle? / ¿Qué nombre quieres ponerle?	5	5
Envía un mensaje de texto.	¿A quién?	¿eh?	¿A quién? / ¿A quién?	5	5
Agrega algo a mi lista de compras.	No veo ninguna app para eso. Tendrás que descargarte una.	¿eh?	No veo ninguna app para eso. Tendrás que descargarte una. / No veo ninguna app para eso. Tendrás que descargarte una.	8	8
Empieza una llamada.	¿A quién?	¿eh?	¿A quién? / ¿A quién?	5	5

Empieza una llamada de vídeo.	¿A quién?	¿eh?	¿A quién? / ¿A quién?	5	5
Crea un listado.	Mis habilidades actuales no llegan a tanto.	¿eh?	Mis habilidades actuales no llegan a tanto. / Mis habilidades actuales no llegan a tanto.	4	4
Envía un correo electrónico.	Por lo que veo no tienes la app mobilemail. Puedes buscarla en el app store.	¿eh?	Por lo que veo no tienes la app mobilemail. Puedes buscarla en el app store. / Por lo que veo no tienes la app mobilemail. Puedes buscarla en el app store.	8	8
**Task 2 Google English**					
**Request - User**	**Response 1 - Assistant**	**Repair Initiator - User**	**Response 2 & 3 – Assistant**	**Machine Repair Strategy 1**	**Machine Repair Strategy 2**
Set an alarm.	Ok, set it for when?	huh?	Sorry, what's the day and time? / [Gives instructions on how to use assistant].	5	9
Set a reminder.	Ok, what's the reminder?	huh?	[No response]. / [No response].	4	4
Set a timer.	Ok, for how long?	huh?	[No response]. /[No response].	4	4
Add something to my calendar.	Ok, what's the title of the event?	huh?	[No response]. / [No response].	4	4
Send a text message.	Ok, who do you want to text?	huh?	Sorry, who do you want to text? / [No response].	5	4
Add to my shopping list.	What do you want to add?	huh?	[No response]. / [No response].	4	4
Make a call.	Ok, which list do you want to add to [Opens notes app]?	huh?	[No response]. / [No response].	4	4
Start a video chat.	What do you want to add?	huh?	[No response]. / [No response].	4	4
Start a list.	Alright, what do you want to call it?	huh?	[No response]. / [No response].	4	4

Send an email.	Who do you want to email?	huh?	[No response]. / Sorry, who do you want to email?	4	5
**Task 2 Google Spanish**					
**Request - User**	**Response 1 - Assistant**	**Repair Initiator - User**	**Response 2 & 3 – Assistant**	**Machine Repair Strategy 1**	**Machine Repair Strategy 2**
Pon una alarma.	Vale, ¿a qué hora la pongo?	¿eh?	[No response]. / [No response].	4	4
Configura un recordatorio.	[Gives instructions on how to use assistant].	¿eh?	[No response]. / [No response].	4	4
Pon un temporizador.	[Gives instructions on how to use assistant].	¿eh?	[No response]. / [No response].	4	4
Agrega algo a mi calendario.	¿Cómo quieres que se llame el evento?	¿eh?	[No response]. / [No response].	4	4
Manda un mensaje de texto.	[Gives instructions on how to use assistant].	¿eh?	[No response]. / [No response].	4	4
Agrega algo a mi lista de compras.	[Gives instructions on how to use assistant].	¿eh?	[No response]. / [No response].	4	4
Empieza una llamada.	¿A quién quieres llamar?	¿eh?	[No response]. / [No response].	4	4
Empieza una llamada de vídeo.	¿A quién quieres hacer una videollamada?	¿eh?	[No response]. / [No response].	4	4
Crea un listado.	[No response, unintelligible phrase partially parsed].	¿eh?	[No response]. / [No response].	4	4
Manda un correo electrónico.	Lo siento, no puedo enviar correos electrónicos en este dispositivo.	¿eh?	[No response]. / [No response].	4	4

Supplementary Table 3. Task C Dialogues in English / Spanish

Sample Dialogues - Task A - English	
**Strategy**	**Utterance**
**1A**	**User**: I have an appointment at the dentist for *{unintelligible phrase}*.<br>**Assistant**: I didn't find any appointment at "dentist" in the next three months.
**1B**	**User**: Are there any *{unintelligible phrase}* in Barcelona?<br>**Assistant**: According to Wikipedia, Camp Nou a stadium in Barcelona, Spain.
**2A**	**User**: How can I get to the street [*unintelligible phrase*]?<br>**Assistant**: I could not find any places matching "Street tata".
**2B**	**User**: Send a text message to Adrian and Sofia that says the following: *{unintelligible phrase}*.<br>**Assistant**: [Creates text that says "the following" and asks for confirmation to send].
**3A**	**User**: Play the song *{unintelligible phrase}*.<br>**Assistant**: Uh oh, there's a problem. Please, try again.
**3B**	**User**: Are there any *{unintelligible phrase}* in Barcelona?<br>**Assistant**: I didn't get that. Could you try again?.
**4A**	**User**: How long does it take from Balmes street to *{unintelligible phrase}*?<br>**Assistant**: Sorry, I can't give you traffic to that location.
**4B**	**User**: What's the duration of the song entitled *{unintelligible phrase}*?<br>**Assistant**: I'm sorry. I can't provide song durations.
**5A**	**User**: Send a message to *{unintelligible phrase}*.<br>**Assistant**: Who do you want to send it to?

**5B**	**User**: Set an appointment for the dentist at *{unintelligible phrase}*.<br>**Assistant**: Alright, when is the event?
**6A**	**User**: What is the capital of *{unintelligible phrase}*?<br>**Assistant**: I found this on the web.
**6B**	**User**: How many days are there in a *{unintelligible phrase}*?<br>**Assistant**: I looked online and found this information that might be helpful.
**7A**	**User**: How can I get to the street *{unintelligible phrase}*?<br>**Assistant**: The best way to get to [displays **user**'s home address] is...
**7B**	**User**: How long does it take from Balmes street to *{unintelligible phrase}*?<br>**Assistant**: [Tells time to get to Blames street from home location].
**8A**	**User**: Send a message to *{unintelligible phrase}*.<br>**Assistant**: Which one do you want to use? [Presents installed apps used for texting].
**8B**	**User**: Play the new album of *{unintelligible phrase}*.<br>**Assistant**: [Opens YouTube app].
**9A**	**User**: *{unintelligible phrase}*.<br>**Assistant**: [Gives instructions about possible actions with the **assistant**].
**9B**	**User**: Could you *{unintelligible phrase}*?<br>**Assistant**: [Provides the website for the **assistant** to learn which commands are possible].
**10A**	**User**: Can you please *{unintelligible phrase}*?<br>**Assistant**: [Attempts to transcribe the *unintelligible phrase*].
**10B**	**User**: I need a *{unintelligible phrase}*.<br>**Assistant**: [Attempts to transcribe the *unintelligible phrase*].

Sample Dialogues - Task B - English	
**Strategy**	**Utterance**
**1A**	**User**: Add to my calendar a doctor's appointment for the *{unintelligible phrase}*.<br>**Assistant**: For what date and time did you want this?<br>**User**: Huh?<br>**Assistant**: I'll add a doctor's appointment entry to your calendar now.
**1B**	**User**: Bring up my playlist with *{unintelligible phrase}* on it.<br>**Assistant**: Which playlist did you want?<br>**User**: Huh?<br>**Assistant**: I will bring up your playlists now.
**2A**	**User**: Send an email.<br>**Assistant**: To whom shall I send it?<br>**User**: Huh?<br>**Assistant**: I couldn't find "huh" in your contacts. To whom shall I send it?
**2B**	**User**: Add something to my calendar.<br>**Assistant**: Ok, what's the title of the event?<br>**User**: Huh?<br>**Assistant**: [Adds event titled "huh"].
**3A**	**User**: Add something to my calendar.<br>**Assistant**: What date and time is your appointment?<br>**User**: Huh?<br>**Assistant**: Uh oh, I missed what you said.
**3B**	**User**: Send a text message.<br>**Assistant**: Who do you want to send it to?<br>**User**: Huh?<br>**Assistant**: I didn't get that. Could you try again?
**4A**	**User**: Set an alarm.<br>**Assistant**: Ok, when's the alarm?<br>**User**: Huh?<br>**Assistant**: No problem, no new alarm set.
**4B**	**User**: Set a timer.<br>**Assistant**: Ok, for how long?<br>**User**: Huh?<br>**Assistant**: I'm still not sure what you mean so I canceled the timer.
**5A**	**User**: Add something to my calendar.<br>**Assistant**: What date and time is your appointment?<br>**User**: Huh?<br>**Assistant**: What date and time is your appointment?

**5B**	**User**: Set a timer.<br>**Assistant**: Ok, for how long?<br>**User**: Huh?<br>**Assistant**: Sorry, for how long?
**6A**	**User**: Add to my calendar a doctor's appointment for the *{unintelligible phrase}*.<br>**Assistant**: For what date and time did you want this?<br>**User**: Huh?<br>**Assistant**: I found this on the web about how to get a doctor’s appointment.
**6B**	**User**: Bring up my playlist with *{unintelligible phrase}* on it.<br>**Assistant**: Which playlist did you want?<br>**User**: Huh?<br>**Assistant**: I was able to find this webpage with information about playlists.
**7A**	**User**: What is the shortest route to Route 66 from my work?<br>**Assistant**: Would you like the shortest distance?<br>**User**: Huh?<br>**Assistant**: [Begins navigating from the **user**’s home address to Route 66].
**7B**	**User**: Show which birthdays I have in my calendar.<br>**Assistant**: Whose birthday did you want to view?<br>**User**: Huh?<br>**Assistant**: [Brings up information about the **user**’s own birthday].
**8A**	**User**: Show which birthdays I have in my calendar.<br>**Assistant**: Whose birthday did you want to view?<br>**User**: Huh?<br>**Assistant**: [Brings up information about the **user**’s own birthday].
**8B**	**User**: I want to see the photo that I took for mom’s birthday.<br>**Assistant**: Which photo are you referring to?<br>**User**: Huh?<br>**Assistant**: [Opens photo gallery application].
**9A**	**User**: Set an alarm.<br>**Assistant**: Set the alarm for when?<br>**User**: Huh?<br>**Assistant**: I’m here to help, get to know more about how to use this assistant at this website [presents assistant’s website].
**9B**	**User**: Set a timer.<br>**Assistant**: For how long?<br>**User**: Huh?<br>**Assistant**: I’m here to help, get to know more about how to use this assistant at this website [presents assistant’s website].

**10A**	**User**: Can you find out how old *{unintelligible phrase}* is?<br>**Assistant**: You mean [attempts to transcribe the *unintelligible phrase*]?<br>**User**: Huh?<br>**Assistant**: [Attempts to transcribe the *unintelligible phrase* again].
**10B**	**User**: Let me see how many people live in *{unintelligible phrase}*.<br>**Assistant**: Are you referring to [attempts to transcribe the *unintelligible phrase*]?<br>**User**: Huh?<br>**Assistant**: [Attempts to transcribe the *unintelligible phrase* again].

Sample Dialogues - Task A - Spanish

Strategy	Utterance
**1A**	**Usuario**: ¿Dónde hay una *{frase no inteligible}* cerca?<br>**Asistente**: ¿Qué tipo de negocio estás buscando?
**1B**	**Usuario**: ¿Hay algún *{frase no inteligible}* en Barcelona?<br>**Asistente**: Según Wikipedia, el estadio de Camp Nou está en Barcelona en España.
**2A**	**Usuario**: ¿Cómo puedo llegar a la calle *{frase no inteligible}*?<br>**Asistente**: Lo siento, no he encontrado calle tata.
**2B**	**Usuario**: Manda un mensaje de texto a María que dice lo siguiente: *{frase no inteligible}*.<br>**Asistente**: [Crea un mensaje de texto con la frase "lo siguiente" y pide confirmación para mandarlo].
**3A**	**Usuario**: Pon la canción *{frase no inteligible}*.<br>**Asistente**: Lo siento, no sé qué quieres que cambie exactamente.
**3B**	**Usuario**: *{frase no inteligible}*.<br>**Asistente**: Lo siento, no te he oído bien. ¿Puedes repetirme lo que has dicho?
**4A**	**Usuario**: ¿Cuál es la duración de la canción que se llama *{frase no inteligible}*?<br>**Asistente**: Lo siento, no te puedo dar la duración de una canción.
**4B**	**Usuario**: ¿Cuánto tiempo tarda viajar a la ciudad que se llama *{frase no inteligible}*?<br>**Asistente**: Lo siento, no te puedo dar el tiempo del viaje.
**5A**	**Usuario**: Tengo una cita en el dentista para *{frase no inteligible}*.<br>**Asistente**: ¿Para qué día es la cita?
**5B**	**Usuario**: Llama a *{frase no inteligible}*.<br>**Asistente**: ¿A quién quieres llamar?

**6A**	**Usuario**: ¿Cuál es la capital de *{frase no inteligible}*?<br>**Asistente**: Vale, esto es lo que he encontrado en internet sobre "¿Cuál es la capital de?". Échale un vistazo.
**6B**	**Usuario**: ¿Cómo puedo llegar a la calle *{frase no inteligible}*?<br>**Asistente**: Aquí tienes unos resultados [busca la palabra "calle" en el internet].
**7A**	**Usuario**: ¿Cuál es el pronóstico de hoy en *{frase no inteligible}*?<br>**Asistente**: Ahora mismo hay algunas nubes y la temperatura es de nueve grados.
**7B**	**Usuario**: ¿Dónde hay una *{frase no inteligible}* cerca?<br>**Asistente**: [Muestra un mapa de la ubicación actual del usuario].
**8A**	**Usuario**: Pon la canción *{frase no inteligible}*.<br>**Asistente**: [Abre la aplicación de YouTube].
**8B**	**Usuario**: Reproduce el nuevo álbum de *{frase no inteligible}*.<br>**Asistente**: [Abre la aplicación de YouTube].
**9A**	**Usuario**: Envía un mensaje a *{frase no inteligible}*.<br>**Asistente**: [Busca en el internet cómo mandar un mensaje usando el sistema del asistente].
**9B**	**Usuario**: Envía un mensaje de texto a María que dice lo siguiente: *{frase no inteligible}*.<br>**Asistente**: [Da instrucciones de cómo pedirle mandar un mensaje].
**10A**	**Usuario**: *{frase no inteligible}*.<br>**Asistente**: [Sin respuesta, el asistente intenta transcribir la *frase no inteligible*].
**10B**	**Usuario**: Quiero escuchar una canción de *{frase no inteligible}*.<br>**Asistente**: [Sin respuesta, el asistente intenta transcribir la *frase no inteligible*].

Sample Dialogues - Task B - Spanish

Strategy	Utterance
**1A**	**Usuario**: Agrega algo a mi lista de compras.<br>**Asistente**: ¿Qué quieres añadir?<br>**Usuario**: ¿Eh?<br>**Asistente**: [Muestra la lista que se llama "compras"].
**1B**	**Usuario**: Busca una lista de reproducción que tiene el artista *{frase no inteligible}*.<br>**Asistente**: ¿Cuál lista de reproducción querías?<br>**Usuario**: ¿Eh?<br>**Asistente**: Mostrando todas tus listas de reproducción ahora.

**2A**	**Usuario**: Manda un correo electrónico.<br>**Asistente**: ¿A quién le debería mandar?<br>**Usuario**: ¿Eh?<br>**Asistente**: No he podido encontrar el contacto “eh” en tu lista de contactos.
**2B**	**Usuario**: Agrega un evento a mi calendario.<br>**Asistente**: Vale, ¿cómo se llama la entrada?<br>**Usuario**: ¿Eh?<br>**Asistente**: [Agrega un evento con el nombre de "eh"].
**3A**	**Usuario**: Pon una alarma.<br>**Asistente**: ¿Para qué hora?<br>**Usuario**: ¿Eh?<br>**Asistente**: No te he entendido. ¿Me lo puedes repetir?
**3B**	**Usuario**: Envía un mensaje de texto.<br>**Asistente**: ¿A quién quieres enviárselo?<br>**Usuario**: ¿Eh?<br>**Asistente**: Lo siento, no he entendido bien. ¿Puedes repetirme lo que has dicho?
**4A**	**Usuario**: Crea un listado.<br>**Asistente**: Lo siento, no puedo ayudarte con eso. Dime si quieres que abra la app “Recordatorios”.<br>**Usuario**: ¿Eh?<br>**Asistente**: Lo siento, no puedo ayudarte con eso.
**4B**	**Usuario**: Empieza un temporizador.<br>**Asistente**: Ok, ¿de cuánto tiempo?<br>**Usuario**: ¿Eh?<br>**Asistente**: No te he entendido bien, así que voy a cancelar el temporizador.
**5A**	**Usuario**: Configura un recordatorio.<br>**Asistente**: ¿Qué quieres que te recuerde?<br>**Usuario**: ¿Eh?<br>**Asistente**: Lo siento, no he entendido bien lo que has dicho. ¿Qué quieres que te recuerde?
**5B**	**Usuario**: Pon un temporizador.<br>**Asistente**: ¿De cuánto tiempo?<br>**Usuario**: ¿Eh?<br>**Asistente**: ¿De cuánto tiempo?
**6A**	**Usuario**: Adiciona a mi calendario la cita con el doctor que se llama Doctor *{frase no inteligible}*.<br>**Asistente**: ¿Para cuál fecha y hora?<br>**Usuario**: ¿Eh?<br>**Asistente**: He encontrado esto en el internet sobre cómo agendar una cita con un doctor.
**6B**	**Usuario**: Muestra mi lista de reproducción con el artista que se llama *{frase no inteligible}*.<br>**Asistente**: ¿Cuál lista de reproducción quieres ver?<br>**Usuario**: ¿Eh?<br>**Asistente**: Encontré esta página web con información sobre las listas de reproducción.

**7A**	**Usuario**: ¿Cuál es la vía más corta entre mi trabajo y la autopista en Castelló?<br>**Asistente**: ¿Quieres ver la distancia más corta?<br>**Usuario**: ¿Eh?<br>**Asistente**: [Empieza a navegar una ruta con destino a la autopista, usando la dirección de la casa del **usuario**].
**7B**	**Usuario**: Quiero ver la foto que tomé de mi mamá.<br>**Asistente**: ¿A cuál te refieres?<br>**Usuario**: ¿Eh?<br>**Asistente**: [Abre el calendario con información sobre el usuario, y no sobre la mamá].
**8A**	**Usuario**: ¿Cómo puedo llegar al puesto de gasolina más cercano?<br>**Asistente**: ¿Cómo quieres llegar?<br>**Usuario**: ¿Eh?<br>**Asistente**: [Abre la aplicación de mapas].
**8B**	**Usuario**: Quiero ver el vídeo que he tomado hoy.<br>**Asistente**: ¿A cuál vídeo te refieres?<br>**Usuario**: ¿Eh?<br>**Asistente**: [Abre la aplicación de la galería de fotos].
**9A**	**Usuario**: Empieza un temporizador.<br>**Asistente**: Ok, ¿de cuánto tiempo?<br>**Usuario**: ¿Eh?<br>**Asistente**: Puedes buscar más información sobre cómo crear un temporizador en [el asistente da el enlace del sitio web para las instrucciones de cómo usarlo].
**9B**	**Usuario**: Borra todas mis alarmas.<br>**Asistente**: Muy bien, ¿cuántas alarmas quieres borrar?<br>**Usuario**: ¿Eh?<br>**Asistente**: Puedes buscar más información sobre cómo programar una alarma en [el **asistente** da el enlace del sitio web para las instrucciones de cómo usarlo].
**10A**	**Usuario**; Configura un recordatorio.<br>**Asistente**: ¿Cuál es el recordatorio?<br>**Usuario**: ¿Eh?<br>**Asistente**: [Sin respuesta, el asistente intenta transcribir la palabra “eh”].
**10B**	**Usuario**: Manda un mensaje de texto.<br>**Asistente**: ¿A quién?<br>**Usuario**: ¿Eh?<br>**Asistente**: [Sin respuesta, el asistente intenta transcribir la palabra “eh”].

Supplementary Table 4. Task C Regression Results - English Task A

English Task A Regression Results							
**Effect**	**Coefficient**	**Std. Error**	**Z Value**	**P Value**	**Odds Ratio**	**CI Lower**	**CI Upper**
**Strategy 5**	1.85	0.27	6.90	<0.001***	6.37	3.76	10.77
**Strategy 3**	1.23	0.26	4.76	<0.001***	3.44	2.07	5.72
**Strategy 8**	0.62	0.25	2.52	<0.05*	1.86	1.15	3.02

**Strategy 2**	0.48	0.26	1.86	0.06	1.62	0.98	2.69
**Strategy 10**	0.47	0.25	1.89	0.06	1.60	0.98	2.59
**Strategy 9**	0.39	0.24	1.59	0.11	1.47	0.91	2.37
**Siri**	0.33	0.11	2.92	<0.01**	1.40	1.12	1.75
**Strategy 6**	0.14	0.24	0.58	0.56	1.15	0.71	1.86
**Strategy 1**	0.00	*Baseline*					
**Strategy 7**	-0.04	0.25	-0.18	0.86	0.96	0.58	1.56
**Strategy 4**	-0.34	0.25	-1.34	0.18	0.71	0.44	1.17

Supplementary Table 5. Task C Regression Results - English Task B

English Task B Regression Results							
**Effect**	**Coefficient**	**Std. Error**	**Z Value**	**P Value**	**Odds Ratio**	**CI Lower**	**CI Upper**
**Strategy 5**	1.99	0.32	6.29	<0.001***	7.29	3.92	13.53
**Strategy 3**	1.28	0.28	4.52	<0.001***	3.59	2.06	6.25
**Strategy 8**	0.89	0.27	3.27	<0.01**	2.43	1.43	4.13
**Strategy 2**	0.46	0.27	1.70	0.09	1.59	0.93	2.71
**Strategy 1**	0.00	*Baseline*					
**Strategy 10**	-0.14	0.26	-0.55	0.58	0.87	0.52	1.45
**Siri**	-0.14	0.12	-1.15	0.25	0.87	0.69	1.10
**Strategy 4**	-0.46	0.26	-1.79	0.07	0.63	0.38	1.05
**Strategy 9**	-0.80	0.26	-3.10	<0.01**	0.45	0.27	0.74
**Strategy 7**	-1.16	0.26	-4.39	<0.001***	0.31	0.19	0.53
**Strategy 6**	-1.61	0.26	-6.09	<0.001***	0.20	0.12	0.34

Supplementary Table 6. Task C Regression Results - English Task A & B Combined

English Task A & B Combined Regression Results							
**Effect**	**Coefficient**	**Std. Error**	**Z Value**	**P Value**	**Odds Ratio**	**CI Lower**	**CI Upper**
**Strategy 5**	1.87	0.20	9.27	<0.001***	6.50	4.38	9.66
**Strategy 3**	1.24	0.19	6.55	<0.001***	3.47	2.39	5.03
**Strategy 8**	0.73	0.18	4.03	<0.001***	2.08	1.46	2.97
**TaskB**	0.51	0.08	6.14	<0.001***	1.66	1.41	1.95
**Strategy 2**	0.48	0.19	2.57	<0.05*	1.61	1.12	2.33
**Strategy 10**	0.18	0.18	1.03	0.30	1.20	0.85	1.71
**Siri**	0.11	0.08	1.35	0.18	1.12	0.95	1.31
**Strategy 1**	0.00	*Baseline*					
**Strategy 9**	-0.17	0.18	-0.98	0.33	0.84	0.59	1.19
**Strategy 4**	-0.36	0.18	-1.98	<0.05*	0.70	0.49	1.00
**Strategy 7**	-0.56	0.18	-3.08	<0.01**	0.57	0.40	0.82
**Strategy 6**	-0.67	0.18	-3.76	<0.001***	0.51	0.36	0.72

Supplementary Table 7. Task C Regression Results – Spanish Task A

Spanish Task A Regression Results

Effect	Coefficient	Std. Error	Z Value	P Value	Odds Ratio	CI Lower	CI Upper
**Strategy 5**	2.12	0.27	7.79	<0.001***	8.31	4.88	14.15
**Strategy 3**	1.10	0.29	3.86	<0.001***	3.01	1.72	5.26
**Strategy 7**	0.77	0.25	3.04	<0.01**	2.15	1.31	3.52
**Siri**	0.43	0.12	3.70	<0.001***	1.53	1.22	1.92
**Strategy 4**	0.21	0.25	0.84	0.40	1.23	0.76	2.02
**Strategy 8**	0.09	0.25	0.37	0.71	1.10	0.68	1.78
**Strategy 1**	0.00	*Baseline*					
**Strategy 2**	-0.10	0.26	-0.38	0.71	0.91	0.55	1.50
**Strategy 10**	-0.41	0.26	-1.59	0.11	0.67	0.40	1.10
**Strategy 9**	-0.99	0.26	-3.84	<0.001***	0.37	0.22	0.62
**Strategy 6**	-1.61	0.26	-6.12	<0.001***	0.20	0.12	0.33

Supplementary Table 8. Task C Regression Results – Spanish Task B

Spanish Task B Regression Results							
**Effect**	**Coefficient**	**Std. Error**	**Z Value**	**P Value**	**Odds Ratio**	**CI Lower**	**CI Upper**
**Strategy 5**	2.24	0.32	7.06	<0.001***	9.36	5.03	17.41
**Strategy 3**	1.67	0.28	5.88	<0.001***	5.34	3.06	9.33
**Siri**	0.45	0.12	3.72	<0.001***	1.56	1.23	1.97
**Strategy 8**	0.11	0.25	0.42	0.67	1.11	0.68	1.80
**Strategy 1**	0.00	*Baseline*					
**Strategy 4**	-0.63	0.26	-2.44	<0.05*	0.53	0.32	0.88
**Strategy 2**	-0.92	0.26	-3.55	<0.001***	0.40	0.24	0.66
**Strategy 7**	-1.65	0.26	-6.25	<0.001***	0.19	0.11	0.32
**Strategy 10**	-1.72	0.26	-6.69	<0.001***	0.18	0.11	0.30
**Strategy 9**	-1.75	0.25	-6.89	<0.001***	0.17	0.11	0.29
**Strategy 6**	-2.22	0.26	-8.62	<0.001***	0.11	0.07	0.18

Supplementary Table 9. Task C Regression Results – Spanish Task A & B Combined

Spanish Task A & B Combined Regression Results							
**Effect**	**Coefficient**	**Std. Error**	**Z Value**	**P Value**	**Odds Ratio**	**CI Lower**	**CI Upper**
**Strategy 5**	2.08	0.20	10.32	<0.001***	7.97	5.37	11.82
**Strategy 3**	1.38	0.20	7.02	<0.001***	3.96	2.70	5.82
**TaskB**	0.49	0.08	5.90	<0.001***	1.63	1.38	1.91
**Siri**	0.41	0.08	4.97	<0.001***	1.51	1.28	1.77
**Strategy 8**	0.06	0.17	0.37	0.71	1.07	0.76	1.50
**Strategy 1**	0.00	*Baseline*					
**Strategy 4**	-0.20	0.18	-1.14	0.26	0.82	0.58	1.16
**Strategy 7**	-0.34	0.18	-1.86	0.06	0.71	0.50	1.02
**Strategy 2**	-0.50	0.18	-2.76	<0.01**	0.61	0.43	0.87
**Strategy 10**	-1.04	0.18	-5.77	<0.001***	0.35	0.25	0.50
**Strategy 9**	-1.34	0.18	-7.49	<0.001***	0.26	0.18	0.37
**Strategy 6**	-1.87	0.18	-10.27	<0.001***	0.15	0.11	0.22

Supplementary Table 10. Task C Regression Results – English & Spanish Totals Combined

**English & Spanish Combined Regression Results**							
**Effect**	**Coefficient**	**Std. Error**	**Z Value**	**P Value**	**Odds Ratio**	**CI Lower**	**CI Upper**
**Strategy 5**	1.97	0.14	13.79	<0.001***	7.19	5.43	9.52
**Strategy 3**	1.28	0.14	9.34	<0.001***	3.58	2.74	4.68
**TaskB**	0.49	0.06	8.43	<0.001***	1.64	1.46	1.83
**Strategy 8**	0.38	0.13	3.05	<0.01**	1.46	1.15	1.87
**Siri**	0.25	0.06	4.38	<0.001***	1.29	1.15	1.45
**Strategy 1**	0.00	*Baseline*					
**Strategy 2**	-0.02	0.13	-0.17	0.87	0.98	0.76	1.26
**Strategy 4**	-0.27	0.13	-2.16	<0.05*	0.76	0.59	0.98
**Strategy 10**	-0.42	0.13	-3.29	<0.01**	0.66	0.52	0.85
**Strategy 7**	-0.45	0.13	-3.54	<0.001***	0.63	0.49	0.82
**LanguageSpanish**	-0.57	0.06	-9.74	<0.001***	0.57	0.50	0.63
**Strategy 9**	-0.74	0.13	-5.93	<0.001***	0.48	0.37	0.61
**Strategy 6**	-1.25	0.13	-9.83	<0.001***	0.29	0.22	0.37
**English & Spanish Combined Pairwise Comparisons**							
**Effect**	**Coefficient**	**Std. Error**	**Z Value**	**P Value**	**Odds Ratio**	**CI Lower**	**CI Upper**
**Strat1 - Strat10**	0.42	0.13	3.29	<0.05*	1.52	1.18	1.94
**Strat1 - Strat2**	0.02	0.13	0.17	1.00	1.02	0.79	1.32
**Strat1 - Strat3**	-1.28	0.14	-9.34	<0.001***	0.28	0.21	0.36
**Strat1 - Strat4**	0.27	0.13	2.16	0.19	1.31	1.03	1.68
**Strat1 - Strat5**	-1.97	0.14	-13.79	<0.001***	0.14	0.11	0.18
**Strat1 - Strat6**	1.25	0.13	9.83	<0.001***	3.50	2.72	4.49
**Strat1 - Strat7**	0.45	0.13	3.54	<0.01**	1.58	1.23	2.03
**Strat1 - Strat8**	-0.38	0.13	-3.05	<0.05*	0.68	0.53	0.87
**Strat1 - Strat9**	0.74	0.13	5.93	<0.001***	2.10	1.64	2.69
**Strat10 - Strat2**	-0.39	0.13	-3.07	<0.05*	0.67	0.52	0.87
**Strat10 - Strat3**	-1.69	0.14	-12.39	<0.001***	0.18	0.14	0.24
**Strat10 - Strat4**	-0.14	0.13	-1.15	0.76	0.87	0.68	1.11
**Strat10 - Strat5**	-2.39	0.14	-16.68	<0.001***	0.09	0.07	0.12
**Strat10 - Strat6**	0.84	0.13	6.66	<0.001***	2.31	1.80	2.95
**Strat10 - Strat7**	0.04	0.13	0.31	1.00	1.04	0.81	1.33
**Strat10 - Strat8**	-0.80	0.12	-6.41	<0.001***	0.45	0.35	0.58
**Strat10 - Strat9**	0.33	0.12	2.64	0.07	1.39	1.09	1.77
**Strat2 - Strat3**	-1.30	0.14	-9.37	<0.001***	0.27	0.21	0.36
**Strat2 - Strat4**	0.25	0.13	1.96	0.25	1.29	1.00	1.65
**Strat2 - Strat5**	-1.99	0.14	-13.79	<0.001***	0.14	0.10	0.18
**Strat2 - Strat6**	1.23	0.13	9.52	<0.001***	3.42	2.66	4.41
**Strat2 - Strat7**	0.43	0.13	3.33	<0.05*	1.54	1.19	1.99
**Strat2 - Strat8**	-0.40	0.13	-3.17	<0.05*	0.67	0.52	0.86

**Strat2 - Strat9**	0.72	0.13	5.67	<0.001***	2.06	1.60	2.64
**Strat3 - Strat4**	1.55	0.14	11.36	<0.001***	4.70	3.60	6.14
**Strat3 - Strat5**	-0.70	0.15	-4.68	<0.001***	0.50	0.37	0.67
**Strat3 - Strat6**	2.53	0.14	18.25	<0.001***	12.52	9.54	16.42
**Strat3 - Strat7**	1.73	0.14	12.50	<0.001***	5.64	4.30	7.40
**Strat3 - Strat8**	0.89	0.13	6.67	<0.001***	2.45	1.88	3.18
**Strat3 - Strat9**	2.02	0.14	14.84	<0.001***	7.53	5.77	9.83
**Strat4 - Strat5**	-2.25	0.14	-15.72	<0.001***	0.11	0.08	0.14
**Strat4 - Strat6**	0.98	0.13	7.79	<0.001***	2.66	2.08	3.41
**Strat4 - Strat7**	0.18	0.13	1.43	0.61	1.20	0.94	1.54
**Strat4 - Strat8**	-0.65	0.12	-5.26	<0.001***	0.52	0.41	0.66
**Strat4 - Strat9**	0.47	0.12	3.80	<0.01**	1.60	1.26	2.04
**Strat5 - Strat6**	3.22	0.15	22.19	<0.001***	25.14	18.91	33.42
**Strat5 - Strat7**	2.43	0.14	16.75	<0.001***	11.33	8.53	15.05
**Strat5 - Strat8**	1.59	0.14	11.35	<0.001***	4.91	3.73	6.47
**Strat5 - Strat9**	2.72	0.14	19.02	<0.001***	15.13	11.43	20.01
**Strat6 - Strat7**	-0.80	0.13	-6.25	<0.001***	0.45	0.35	0.58
**Strat6 - Strat8**	-1.63	0.13	-12.98	<0.001***	0.20	0.15	0.25
**Strat6 - Strat9**	-0.51	0.12	-4.09	<0.001***	0.60	0.47	0.77
**Strat7 - Strat8**	-0.84	0.13	-6.61	<0.001***	0.43	0.34	0.56
**Strat7 - Strat9**	0.29	0.13	2.29	0.15	1.34	1.04	1.71
**Strat8 - Strat9**	1.12	0.12	9.10	<0.001***	3.08	2.42	3.92
